# Enriched Environmental Conditions Modify the Gut Microbiome Composition and Fecal Markers of Inflammation in Parkinson’s Disease

**DOI:** 10.3389/fnins.2019.01032

**Published:** 2019-10-15

**Authors:** Yogesh Singh, Mohamed El-Hadidi, Jakob Admard, Zinah Wassouf, Julia M. Schulze-Hentrich, Ursula Kohlhofer, Leticia Quintanilla-Martinez, Daniel Huson, Olaf Riess, Nicolas Casadei

**Affiliations:** ^1^Institute of Medical Genetics and Applied Genomics, University of Tübingen, Tübingen, Germany; ^2^Algorithms in Bioinformatics, Faculty of Computer Science, University of Tübingen, Tübingen, Germany; ^3^Bioinformatics, Center for Informatics Science, Nile University, Giza, Egypt; ^4^Institute of Pathology, Comprehensive Cancer Center, University Hospital, University of Tübingen, Tübingen, Germany

**Keywords:** Parkinson’s disease, gut microbiota, calprotectin, inflammation, α-Synuclein, 16S rRNA, enriched environment

## Abstract

Recent findings suggest an implication of the gut microbiome in Parkinson’s disease (PD) patients. PD onset and progression has also been linked with various environmental factors such as physical activity, exposure to pesticides, head injury, nicotine, and dietary factors. In this study, we used a mouse model, overexpressing the complete human SNCA gene (SNCA-TG mice) modeling familial and sporadic forms of PD to study whether environmental conditions such as standard *vs.* enriched environment changes the gut microbiome and influences disease progression. We performed 16S rRNA DNA sequencing on fecal samples for microbiome analysis and studied fecal inflammatory calprotectin from the colon of control and SNCA-TG mice kept under standard environment (SE) and enriched environment (EE) conditions. The overall composition of the gut microbiota was not changed in SNCA-TG mice compared with WT in EE with respect to SE. However, individual gut bacteria at genus level such as *Lactobacillus* sp. was a significant changed in the SNCA-TG mice. EE significantly reduced colon fecal inflammatory calprotectin protein in WT and SNCA-TG EE compared to SE. Moreover, EE reduces the pro-inflammatory cytokines in the feces and inflammation inducing genes in the colon. Our data suggest that an enriched social environment has a positive effect on the induction of SNCA mediated inflammation in the intestine and by modulating anti-inflammatory gut bacteria.

## Introduction

Chronic inflammation is a key process in the progression of Parkinson’s disease (PD) (Li et al., 2016). Besides the main pathological features of Lewy bodies (LBs) and neurites predominantly composed of α-Synuclein (α-Syn) protein (Marques and Outeiro, 2012; Wales et al., 2013) in the brain and gut of PD patients, neuroinflammatory markers such as reactive microglial expression of HLA-DR, CXCL2 and S100b in *substantia nigra* (SN), IgG in LBs, proinflammatory cytokines (IL-1β and TNF-α) in SN and striatum are additional neuropathological characteristics of PD (Gelders et al., 2018). Interestingly, although multiple studies showed a progression of the pathology (also termed either α-Synucleinopathy or Synucleinopathy) through the brain (Braak et al., 2003; Visanji et al., 2014), other studies also proposed a possible spread of the disease from neurons to neurons *via* seeding of α-Syn in the peripheral nervous system (Li et al., 2008; Luk et al., 2009). Accordingly, injection of α-Syn in the brain (Luk et al., 2012), in the gut (Holmqvist et al., 2014) or in muscles (Sacino et al., 2014) pointed to a retrograde transport of protein seeds from the peripheral nervous system to the central nervous system. Thus, in addition to the genetic factors, external or unknown internal factors inducing α-Syn aggregation or expression such as environmental conditions, diet or lifestyle, bacterial metabolites and self-antigens may also trigger or enhance PD (Yamin et al., 2003; Bischoff et al., 2014; Klingelhoefer and Reichmann, 2015; Kotloski and Sutula, 2015; Mulak and Bonaz, 2015; Felice et al., 2016; Sulzer et al., 2017; Caputi and Giron, 2018; Erro et al., 2018).

Environmental factors appeared to improve and worsen the pathology of PD (Klingelhoefer and Reichmann, 2015; Delamarre and Meissner, 2017; Erro et al., 2018; Hu et al., 2019). Several chemicals or pesticides such as MPTP (Muthukumaran et al., 2014), paraquat (Park et al., 2005; Barlow et al., 2007), rotenone (Betarbet et al., 2000), viral infections (Bhattacharjee and Lukiw, 2013), etc., exposure to which lead to neurodegenerative effects whilst enriched environmental (EE) conditions based on studies of animal models suggested that enrichment can promote neuronal activation, signaling and plasticity in various brain regions. Therefore, EE conditions could possibly exert neuroprotective effects on neurons and delay pathogenesis in PD (Jadavji et al., 2006; Nithianantharajah and Hannan, 2006; Kotloski and Sutula, 2015).

The gut harbors a dynamic environment and is exposed to environmental factors such as diet, antibiotics, and pathogens and follows on constant interaction with microbial communities (Laukens et al., 2015; Koh et al., 2016; Thaiss et al., 2016). In addition, the gut microbiota is also shaped throughout life by host-related factors such as the host genotype as well as with aging (Blander et al., 2017). Disturbances within gut microbiota have been reported to influence host susceptibility to pathogens and pathological conditions such as gastrointestinal inflammatory diseases and obesity (Ridaura et al., 2013; Ellekilde et al., 2014; Devkota and Chang, 2015; Liu et al., 2017). Gastrointestinal dysfunctions are frequently reported by PD patients (Fasano et al., 2015) and in some clinical studies precede the onset of motor symptoms (Bassotti et al., 2000; Abbott et al., 2001). In this context, the presence of Synucleinopathy in the autonomic nervous system of PD patients (Rao and Gershon, 2016), accumulation of α-Syn in the bowel of PD patients (Hilton et al., 2014) as well as a modified intestinal microbiota correlated to motor symptoms suggest a potential origin of Synucleinopathy in the gastrointestinal tract (GIT) (Scheperjans et al., 2015). Several recent patients from different countries (United States, Japan, China, Germany, and Netherlands) and mouse studies suggest a potential role of the gut microbiome in PD pathogenesis (Keshavarzian et al., 2015; Scheperjans et al., 2015; Sampson et al., 2016; Hopfner et al., 2017; Minato et al., 2017; Pereira et al., 2017; Petrov et al., 2017; Lin et al., 2018; van Kessel et al., 2019) as gut dysbiosis may lead to an inflammatory environment potentially initiating synucleinopathy (Stojkovska et al., 2015; Powell et al., 2017; Proctor et al., 2017).

In this study, we aim to investigate if the expression of α-Syn in the gut of a humanized mouse model of PD can be influenced by EE conditions and explore how EE conditions interact with the gut microbiome to affect the inflammation in the gut.

## Materials and Methods

### Animal Experiments and EE Conditions

C57BL/6N mice were obtained from Charles River and were housed in colony cages under a 12 h light-dark cycle. BAC-hSNCA transgenic (SNCA-TG) mice and EE conditions have been described earlier by our group (Wassouf et al., 2018). Both of the genotype animals (WT and SNCA-TG) were randomly allotted to either the standard environment (SE) or the enriched environment (EE). Only female mice were used for our experimental study as earlier reports highlighted that aggressive behavior occurs between male mice house in a long-term enriched environmental condition (Marashi et al., 2003). For the SE groups (WT and SNCA-TG), three to four mice were housed in standard cages (Type II long). All the SE group animals had free access to normal chow diet and tap water. The EE groups animals (WT and SNCA-TG), type IV cage was used with more bedding and nesting material and in total eight female were co-housed together. To promote enhanced sensory stimulation, enriched cages were additionally provided with objects varying in color, size, shape and texture which were rearranged three times a week in order to maintain novelty and complexity. To improve the physical activity in the EE conditions, type IV cages were equipped with tunnels, climbing cubes, saucer wheels and running wheels (Wassouf et al., 2018). Animals were housed in their respective SE or EE from weaning until the end of the study. To minimize the changes in microbiome diversity due to cage and hormone cycles in female mice, we used WT and SNCA-TG in separate SE and EE cages to collect the feces. To assess the impact of α-Syn and enriched environmental conditions on microbiome composition changes, 12-month-old female mice were used.

### Euthanasia and Sample Collection

To minimize the influence of preparation on the gut microbiome, mice were euthanized *via* cervical dislocation during the light phase. GIT was then prepared rapidly on ice, content of the gut as well as the rest of the tissue rinsed in PBS (10 mM phosphate, 150 mM NaCl, pH 7.4) were collected in cryotubes and snap frozen by liquid N_2_ and stored at −80°C until use.

### DNA Isolation, Preparation, and Sequencing

DNA was isolated from the fecal and cecal contents. Briefly, 10 to 20 mg of digesta was mixed with 180 μl of 20 mg/ml lysozyme (#L6876; Sigma) in 20 mM Tris–HCl at pH 8.0, 2 mM EDTA and 1.2% of triton-X. The suspended pellet was incubated under agitation for 30 min at 37°C and then digested at 56°C for 30 min with 20 μl of 50 mg/ml proteinase K (#315836; Roche) in 200 μl of buffer AL. Digestion was inactivated at 95°C for 15 min. After centrifugation, 200 μl of ethanol was added to the samples prior binding to the QIAamp spin column (#51104; QIAamp DNA Blood mini kit; Qiagen) then samples were centrifuged at 8000 rpm for 1 min. Samples washed successively in 500 μl of buffer AW1 and AW2. DNA eluted from the column in 200 μl of buffer AE and stored at −20°C.

For the 16S rRNA amplification, 12.5 ng of DNA was amplified using 0.2 μM of both forward primer (TCGTCGGCAGCGTCAGATGTGTATAAGAGACAGCCTAC GGGNGGCWGCAG, Metabion) and reverse primer (GTCTC GTGGGCTCGGAGATGTGTATAAGAGACAGGACTACHVG GGTATCTAATCC, Metabion) and KAPA HiFi HotStart Ready Mix (#KK2601; KAPABiosystems, Germany). PCR was performed using a first denaturation of 95°C for min, followed by 25 cycles of amplification at 95°C for 30 s, 55°C for 30 s and 72°C for 30 s, final elongation at 72°C for 5 min and the amplified DNA stored at 4°C. Electrophoresis of samples was used to verify amplicon specificity.

Samples were then purified (#A63881; Agencourt AMPure XP, Beckman Coulter) and PCR amplicons indexed using the Nextera XT index (#FC-131-1096; Nextera XT DNA Library Preparation kit) and KAPA HiFi HotStart ReadyMix. PCR was performed using a first denaturation of 95°C for 3 min, followed by 8 cycles of amplification at 95°C for 30 s, 55°C for 30 s and 72°C for 30, final elongation at 72°C for 5 min. Purified PCR samples were then validated using the BioAnalyzer (Bioanalyzer DNA 1000, Agilent) and 4 nM of each library pooled using unique indices before sequencing on a MiSeq (Illumina) and paired 300-bp reads.

### Sequence Analysis and Statistics

Available sequence data has been trimmed and filtered using SeqPurge (Sturm et al., 2016). Trimming parameters demanded a minimum quality at 3′ end of *q* = 35 (parameter qcut = 35). Processed sequence data were aligned using MALT (version 0.3.8^1^) against the 16S database SILVA SSU Ref Nr 99^2^ and classified using NCBI taxonomy. Alignment was performed using semi-global alignment and a minimum sequence identity of 90% (parameter minPercentIdentity = 90). Further analysis and visualization was performed using MEGAN (MEtaGenome Analyzer) version 6.0 (Huson et al., 2016). In Figure 4C, data were normalized with SE and EE conditions and shown as percentage abundance. Alpha (statistical) was set *a priori* at 0.05 for all tests of significance.

### Preparation of Tissue for Histological Analysis

Gut samples prepared on ice were fixed for 24 h in 4% paraformaldehyde (PFA), stored at 4°C in 0.4% PFA for a maximum of 4 weeks prior to embedding in paraffin. Brain samples were prepared from mice anesthetized by CO_2_ inhalation and transcardially perfused with PBS and cold 4% PFA diluted in PBS. Brains were removed carefully from the skull and post-fixed 24 h in 4% PFA. Fixed samples were then alcohol-dehydrated and embedded in paraffin blocks using a tissue embedding station and stored at room temperature until use. Paraffin blocks containing colon and other GIT tissues were cut into 7 μm thick sections using a microtome, placed in 45°C water bath for flattening then collected onto a glass slide, dried in an incubator at 50°C for 1 h and stored at room temperature.

### Immunohistochemistry

Immunohistochemistry (IHC) was performed on colon sections as described earlier on brain sections (Nuber et al., 2013) using additional antibodies (human and mouse Anti-α-Syn: #610786 (clone MC42) BD Biosciences; human Anti-α-Syn: #ab27766 (LB509) Abcam, human Anti-α-Syn: #ALX-804-258-L001 (15g7), Enzo Life Sciences; Anti-NeuN, clone A60: #MAB377, Merck Millipore; Anti-MAP2: #ab70218 Abcam; Anti-TH: #657012, Merck Millipore; Anti-GFAP: #Z0334, Dako).

### Tissue Lysate

Gut tissue were weighted frozen and lysed with 10 volumes of RIPA buffer (50 mM Tris, 150 mM NaCl, 1.0% NP-40, 0.5% sodium deoxycholate, 0.1% SDS, pH 8.0) supplemented with protease inhibitor (#11697498001; cOmplete^TM^ Protease Inhibitor Cocktail, Roche Diagnostics). Brain tissues were homogenized for 30 s using a disperser (T10 ULTRA-TURRAX; VWR) on ice. After homogenization, samples were incubated for 30 min at 4°C and spun for 20 min at 12 000 g. Proteins lysate supernatants were supplemented with 10% glycerol before long storage at −80°C. Protein concentrations were determined using BCA method (#23225; Thermo Fisher Scientific, Germany).

### Western Blotting

Samples were prepared by diluting protein lysates in PAGE buffer (0.2 M glycine, 25 mM Tris, 1% SDS), followed by a denaturation at 95°C for 10 min in loading buffer (80 mM Tris, 2% SDS, 5% 2-mercaptoethanol, 10% glycerol, 0.005% bromophenol blue, pH 6.8) and a short centrifugation of 30 s at 400 *g*. Proteins were separated by electrophoresis using 12% SDS-PAGE gel. Gels containing proteins were washed for 5 min in transfer buffer (0.2 M glycine, 25 mM Tris, 10–20% methanol) and transferred to membranes equilibrated in transfer buffer. Transfer was performed for 90 min at 80 V at 4°C on 0.45 μm nitrocellulose membranes (#88018, Thermo Fisher Scientific). Immunoblots were washed for 5 min in TBS buffer and blocked using 5% non-fat milk (Slim Fast) in TBS. Membranes were then washed twice for 5 min in TBST and then were incubated with the primary antibody over night at 4°C (human and mouse α-syn: #610786 BD Biosciences; human α-syn: #804-258-L001, Enzo Life Sciences; β-actin: #A4700, Sigma). After incubation with the first antibody, membranes were washed four times (5 min each) with TBST. Membranes were then incubated for 75 min with the secondary antibody coupled to horseradish peroxidase (#NA931-1ML; Amersham ECL Mouse IgG, HRP linked whole Ab, #NA935; Amersham ECL Rat IgG, HRP-linked whole Ab, both from GE Healthcare Life Sciences). After four washing steps with TBST (5 min each), bands were visualized using the enhanced chemiluminescence method (#RPN2232; ECL^TM^; GE Healthcare Life Sciences). Light signals were detected using LI-COR Odyssey and quantified using Odyssey software.

### Calprotectin ELISA

To measure the calprotectin/MRP 8/14 in the fecal samples, a S100A8/S100A9 ELISA kit (#K6936, Immundiagnostik AG, Germany) was used according to the manufacturer’s guidelines. Fecal samples were measured (weight between 20 mg and 50 mg), dissolved in 500 μl of extraction buffer, mixed then by vortexing and centrifuged for 10 min at 3000 × *g*. The supernatant was transferred to a new microcentrifuge tube and 100 μl of the sample was used for measuring the protein. The data was analyzed using the 4-parameter algorithm and the concentration of calprotectin was normalized with feces weight and data presented in ng/g.

### Mouse Inflammation Panel 13-Plex Cytokine Measurements in Feces by Flow Cytometry

To measure the cytokines, feces were dissolved in 500 μl of extraction buffer, mixed by vortexing and then centrifuged for 10 min at 3000 × *g* (#K6936, Immundiagnostik AG). The supernatant transferred to a new microcentrifuge tube and 25 μl of the sample was used for measuring the protein as described in the manufacturer’s protocol (LEGENDPlex Mouse Inflammation Panel (13-plex) with v-bottom plate #740446; BioLegend, Germany). Standards were prepared as suggested in the kit. In brief, 96 v-bottom plate was used for measuring the cytokines in the feces. 25 μl of capture beads and 25 μl feces supernatant was added to each well and incubated (different size for each cytokine and labeled with APC) for 2 h on a shaker at speed of 800 rpm. After a 2 h incubation, the plate was washed with wash buffer (1×), 25 μl detection antibody added then brought back on the shaker again. After a 1 h further incubation, without washing, 25 μl of SA-PE (biotinylated detection antibody) was added and incubated for 30 min on a shaker again. The plate was centrifuged at 250 × *g* for 5 min, discarded the supernatant and added the 150 μl of wash buffer (1×). Beads were resuspended by pipetting and sample were acquired using Flow cytometry (BD LSRFortessa, BD Biosciences, Germany) for cytokine measurement. The FCS files were generated on a flow cytometer and analyzed using BioLegend’s LEGENDplex data analysis software and cytokines were presented in pg/g.

### RNA-Sequencing From Colon Tissues

Total RNA was extracted using the QIAsymphony RNA kit (#931636, Qiagen). Briefly, 10 to 20 mg of frozen tissue was dissociated using 400 μl of RLT Plus in a 2 ml extraction tube containing 5 mm diameter beads (Qiagen) and agitated at 30 Hz for twice 2 min using TissueLyser II (Qiagen). RNA isolation was performed on the QIAsymphony (Qiagen) following the platform Standard protocol. Elution was performed using 50 μl of RNase-free water.

RNA quality was assessed with an Agilent 2100 Bioanalyzer and the Agilent RNA 6000 Nano kit (#5067-1511; Agilent). 3′ RNA-sequencing was performed using 100 ng of total RNA and the QuantSeq 3′ mRNA-Seq (#015.24; Lexogen, Austria). Libraries were sequenced on the NextSeq500 using the Mid Output v2.5 150 Cycles (#20024904, Illumina) with a depth of >2 millions reads each.

FASTQ were generated using the fastp (v0.20.0) and RNA-seq data quality was assessed to identify any known potential issue with sequencing cycles, low average quality, adaptor contamination, or repetitive sequences from PCR amplification. Reads were aligned using STAR (v2.7.2a) against a custom-built genome composed of the Ensembl Mus musculus grcm38. Alignment quality was analyzed using MappingQC (v1.8) and visually inspected in the Integrative Genome Viewer (v2.4.19). Normalized read counts for all genes were obtained using edgeR (v3.26.6). Transcripts covered with less than 4 count-per-million in at least 1 sample were excluded from the analysis leaving >14,000 genes for determining differential expression in each of the pair-wise comparisons between experimental groups.

RNA sequencing data was further subjected to Ingenuity pathway analysis (IPA) to identify the genes involved in different canonical pathways and further how these genes could be governed by other upstream regulators.

### Statistical Analysis

MEGAN-CE (version 6.10.6, built 20 Dec 2017 and version 6.14.2, built 23 Jan 2019) was used for data acquisition and analysis. GraphPad and Inkscape were used for the final figure preparation. One way ANOVA and student’s *t*-test was used for statistical analysis using GraphPad. The *p*-value (≤0.05) was considered significant.

## Results

### Human α-Syn Expression in the Enteric Nervous System of the Gut in SNCA-TG Mice

**FIGURE 1:**
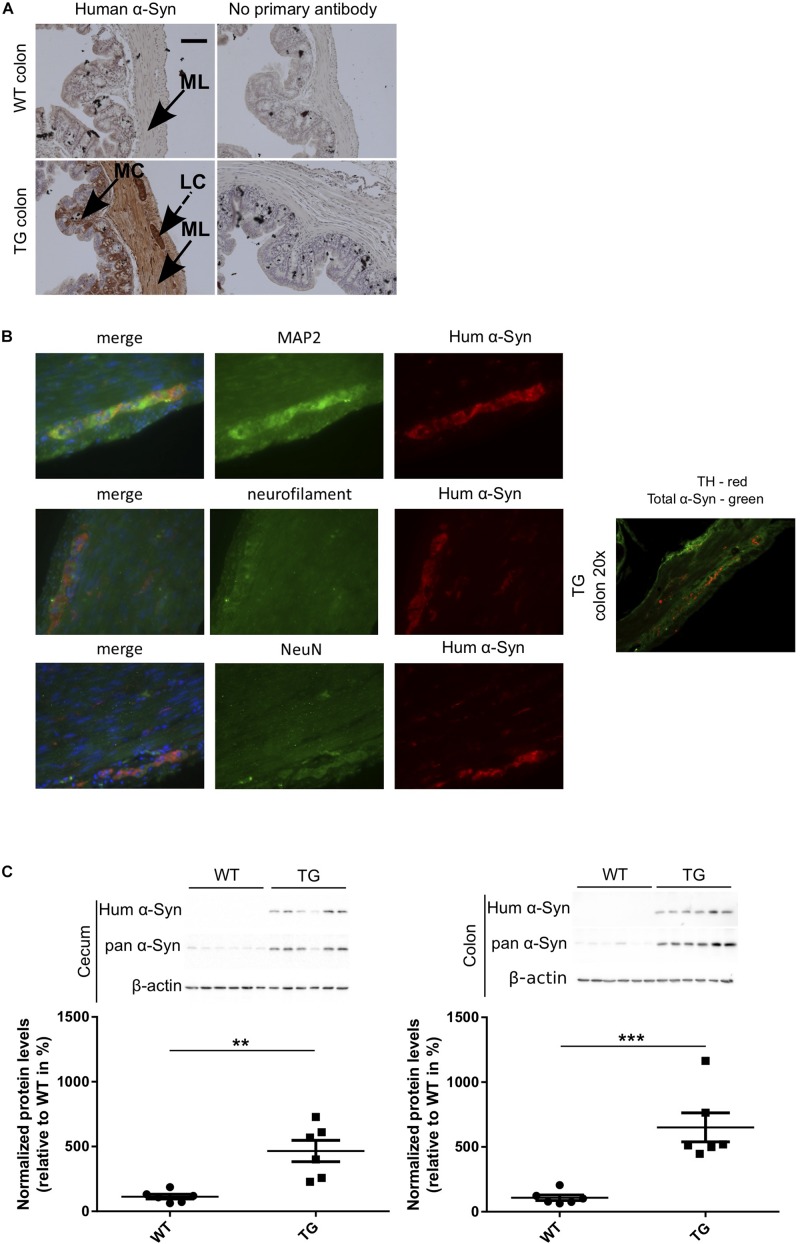
Human α-Syn expression in the ENS (colon) of SNCA-TG (TG) mice. Expression of the transgenic human α-Syn protein was investigated by IHC and Immunoblotting **(A)**. Transgenic human α-Syn protein was detected in the GIT of TG mice using IHC in the muscular layer, longitudinal muscle cells, and mucosal layer of the colon. **(A,B)** Double immunofluorescence staining (SNCA-TG colon) suggests the presence of human α-Syn (15g7 clone human α-syn: #804-258-L001, Enzo Life Sciences as well as MC42 clone human and mouse α-syn: #610786 BD Biosciences) in enteric neurons labeled with the synaptic markers MAP2, the neuronal markers neurofilament (NeuN), and the dopaminergic marker TH (20×). Red and green color-coded staining shows the respective antibodies staining. **(C)** Expression of human α-Syn in the gut was confirmed using Western blotting of 12-month old mice (*n* = 6). Unpaired Student’s two-tailed *t*-test was performed. *p*-Value was considered significant if it was less than or equal to 0.05 (^∗∗^*p* ≤ 0.01, ^∗∗∗^*p* ≤ 0.001). For the purpose of ease in all the artwork (figures), we have denoted SNCA-TG as TG.

To study if the expression of α-Syn induces changes of the gastrointestinal microbiome, we used a SNCA-TG mouse model of PD overexpressing the complete human *SNCA* gene including its promoter, introns, exons and UTRs (Yamakado et al., 2012), as reported previously (Wassouf et al., 2018). This model overexpress the human protein with a spatial distribution in the brain similar to the endogenous human and mouse expression (Wassouf et al., 2018). Using IHC, we investigated the expression of the transgenic α-Syn in the gastrointestinal tract of the SNCA-TG mice and observed presence of the human α-Syn protein in the colon and the entire gut including stomach, ileum, cecum, and rectum, respectively (Figure 1A and Supplementary Figure S1). Staining was observed in the muscular layer and more particularly in the longitudinal muscle. Interestingly, we also observed a specific staining in the mucosal layer of the colon. Double immunofluorescence staining was performed using antibodies against human α-Syn and neuronal markers such as Map2, Neurofilament, and NeuN. Staining identified double-labeled neurons positive for human α-Syn protein and confirms a strong expression of the transgenic protein in the ENS (Figure 1B). A strong expression of human α-Syn was also confirmed using Western blots (Figure 1C). The level of total α-Syn observed in SNCA-TG mice was significantly increased in SNCA-TG mice compared to WT in both, the cecum and the colon (Figure 1C). Thus, these results suggested that our SNCA-TG animal model is suitable to study the role of SNCA overexpression in the gut and to study host-pathogen interactions for the impact of EE conditions on the accumulation of α-Syn.

### Changes in the Gut Microbiome Composition in SNCA-TG Mice Under SE Conditions

**FIGURE 2:**
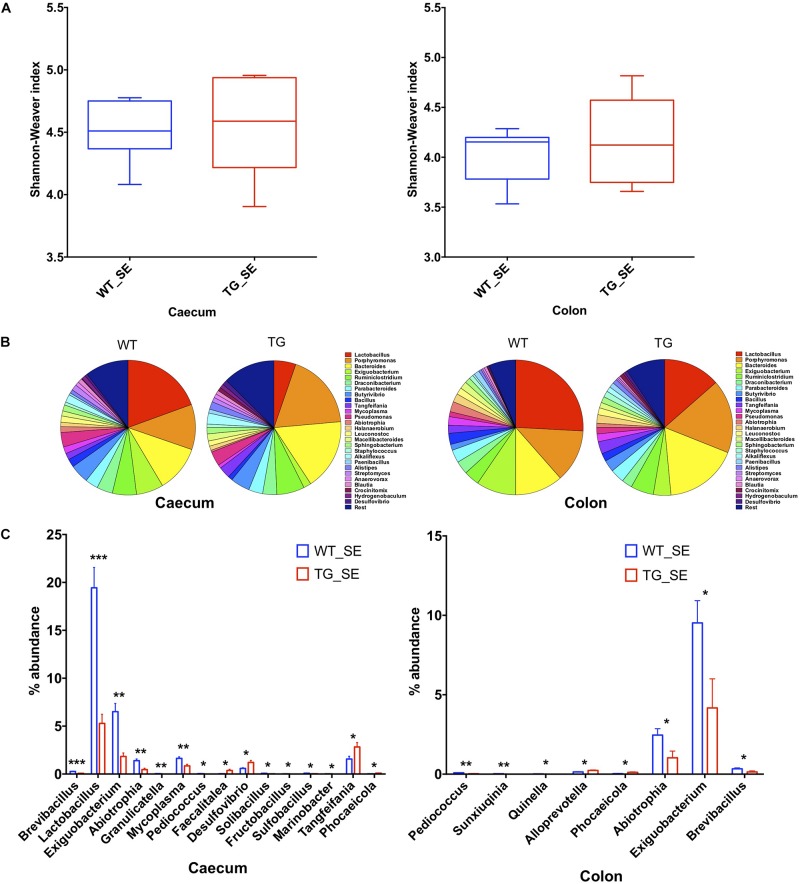
Gut dysbiosis in the cecum and colon of SNCA-TG mice under SE conditions. 16S rRNA sequencing was performed and α-diversity (Shannon–Weaver index) was measured using MEGAN-CE for the cecum and colon. **(A)** Shannon–Weaver index was almost similar in WT and SNCA-TG in either cecum or colon. **(B)** The bacterial abundance at genus level (left figure is for ceacum- WT and TG and right figure for colon – WT and TG) was calculated in percentage in the cecum and colon. Top 1% bacterial abundance is shown here (32/543) genera. **(C)** In the cecum 15 different bacterial genera (*Brevibacillus, Lactobacillus, Exiguobacterium, Abiotrophia, Granulicatella, Mycoplasma, Pediococcus, Faecalitalea, Desufovibrio, Solibacillus, Fructobacillus, Sulfobacillus, Marinobacter, Tangfeifania and Phocaeicola*) were significantly different between WT and SNCA-TG mice. However, in the colon only 8 genera (*Pediococcus, Sunxiuqinia, Quinella, Alloprevotella, Phocaeicola, Abiotrophia, Exiguobacterium, Brevibacillus*) were different in the colon in between WT and SNCA-TG mice. The bacterial genera (*Pediococcus, Phocaeicola, Abiotrophia, Exiguobacterium, Brevibacillus*) were common in the cecum and colon and were significantly different in WT and SNCA-TG mice. Unpaired Student’s two-tailed *t*-test was performed the significance for both the cecum and colon independently. Data are presented in decreasing *p*-values. *p*-Value was considered significant if it was less than or equal to 0.05 (^∗^*p* ≤ 0.05, ^∗∗^*p* ≤ 0.01, ^∗∗∗^*p* ≤ 0.001).

To compare the gut microbial communities diversity, we isolated DNA from 6 and 12 month-old WT and SNCA-TG cecum and colon mouse digesta, performed PCR of the 16S ribosomal RNA (16S rRNA) and sequenced the amplicons using next generation sequencing (Ley et al., 2006; Jovel et al., 2016). Due to the elevated expression of SNCA and the involvement of constipation in the mice overexpressing SNCA (Kuo et al., 2010; Andrews and Storr, 2011), we focused on the cecum and colon microbiota and sequenced both groups with a comparable depth of ∼ 60,000–100,000 clusters per sample from 6 and 12 months (Supplementary Figures S2A, S3A, S4A). We then evaluated the population diversity of the microbial community (alpha-diversity) using the Shannon–Weaver index (SWI) and found no significant differences between the WT and SNCA-TG cecum and colon samples at 6 and 12 months (Figure 2A and Supplementary Figure S4D). Most of the reads (∼90%) represented the Firmicutes and Bacteroidetes, which are typically the dominant phyla in the microbiome and belong to the strict anaerobic bacterial group (Browne et al., 2016; Supplementary Figures S2B, S3B, S4B,C). The phylum Firmicutes was significantly lower in SNCA-TG animals compared with WT (12 months) in the cecum, but no significant difference was observed in the colon (12 and 6 months) (Supplementary Figures S2B, S3B, S4B,C). In both, the cecum and the colon, SNCA-TG mice had lower levels of Firmicutes/Bacteroidetes ratio (FBR) (Supplementary Figures S2B,C, S3B,C), however, no significant difference was observed in the 6-month age group (Supplementary Figure S4).

We then investigated differences in taxa at the genera level among microbial communities (beta-diversity) (Morgan et al., 2013). We used principal component analysis (PCoA) tool of Megan6 (Huson et al., 2016) to investigate SNCA-TG and WT samples based on beta diversity metrics which showed a slight shift in clustering for both the groups in the cecum and colon under SE conditions, respectively both 6 and 12 months age group, respectively (Supplementary Figures S4E, S5A,B). We performed an explorative analysis based on the 28 most abundant bacteria genera (more than 1% abundance) and representing more than ∼88−92% of the total bacteria sequenced in the cecum and the colon (Figure 2C). We identified 15 bacterial genera (*Brevibacillus, Lactobacillus, Exiguobacterium, Abiotrophia, Granulicatella, Mycoplasma, Pediococcus, Faecalitalea, Desulphovibrio, Solibacillus, Fructobacillus, Sulfobacillus, Marinobacter, Tangfeifania, Phocaeicola*; with decreasing order of *p*-values; student’s *t*-test) in the cecum and 8 bacterial genera (*Pediococcus, Sunxiuqinia, Quinella, Phocaeicola, Alloprevotella, Abiotrophia, Exiguobacterium, Brevibacillus*; with decreasing order of *p*-value; student’s *t*-test) in the colon which were differently represented in SNCA-TG mice under SE conditions in the 12-month age group (Figure 2C). In the 6-month age group, only *Anaerotruncus, Helicobacter, Klebsiella, Streptococcus, Ruminococcus, Subdoligranulum* and *Azotobacter* (*p*-value in decreasing order) were significantly different in WT and SNCA-TG mice under SE conditions, however, abundance was only found between that 0.01–0.3 percentage (Supplementary Figure S4F). Most abundantly present genera such as *Lactobacillus, Bacteroidales, Ruminiclostridium*, etc., were not different at all in WT and SNCA-TG (6 months age group) in SE conditions (Supplementary Figures S4G,H), therefore, we did not pursue the EE conditions for further 16s rRNA analysis in the colon and cecum samples.

Five bacterial genera (*Pediococcus, Abiotrophia, Phocaeicola, Exiguobacterium, and Brevibacillus*) were significantly different percentage wise in both the cecum and colon (Figure 2C) of SNCA-TG compared with WT mice (12-month age group). *Lactobacillus* genus was the most abundant in the mouse cecum and colon, thus we explored further for the *Lactobacillus* genus abundance which was significantly reduced in cecum samples of SNCA-TG mice compared with WT (*p* = 0.0001; Student’s *t*-test), and in a similar way *Lactobacillus* was also reduced in the colon of the SNCA-TG mice, although it did not reach significance level (*p* = 0.07; Student’s *t*-test).

### Microbial Diversity Under EE Conditions in SNCA-TG Mice

**FIGURE 3:**
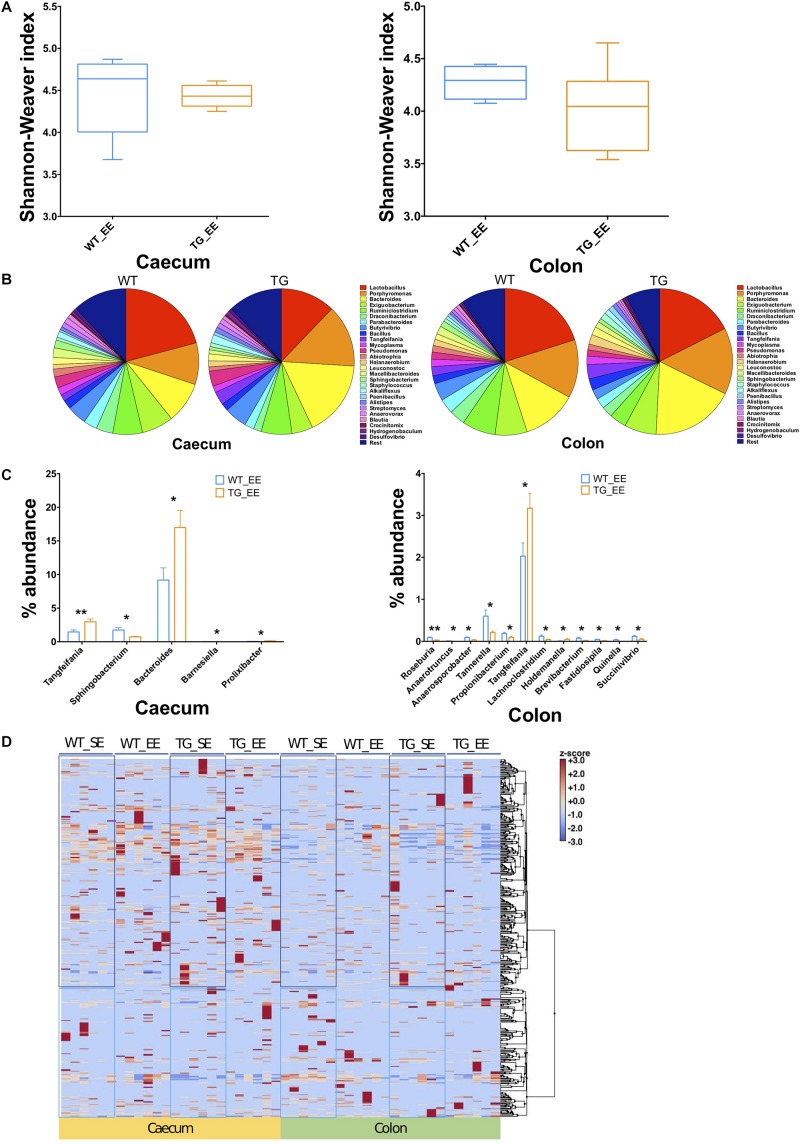
Enriched environment conditions moderate the gut dysbiosis in SNCA-TG mice. In a similar fashion to SE conditions, α-diversity (Shannon–Weaver index) was measured in EE conditions using MEGAN-CE for the cecum and colon. **(A)** Shannon–Weaver index was tending to be lower in SNCA-TG compared with WT in both the cecum and colon. However, it was not significantly different. **(B)** The bacterial abundance at genus level was calculated as a percentage in the cecum (left hand side) and colon (right hand side). **(C)** In EE conditions, levels of the bacterial dysbiosis in the cecum only 5 different bacterial genera (*Tangfeifania, Sphingobacterium, Bacteroides, Barnesiella and Prolixibacter*) were significantly different between WT and SNCA-TG mice. However, 12 genera (*Roseburia, Anaerotruncus, Anaerosporobacter, Tannerella, Propionibacterium, Tangfeifania, Lachnoclostridium, Holdemanella, Brevibacterium, Fastidiosipila, Quinella and Succinivibrio*) were different in the colon for WT and SNCA-TG mice. The only one bacterial genus *Tangfeifania* was common in both the cecum and colon which was significantly different between WT and SNCA-TG mice. Unpaired Student’s two-tailed *t*-test was performed the significance measurements for the cecum and colon independently. **(D)** Overall clustering of bacterial genera is presented a heat-map. Abundance of bacterial presence is shown in *Z*-score (–3.0 to +3.0) values. Two distinct clustering of bacterial genera appeared in both the cecum and colon in EE and SE conditions. Data are presented in decreasing *p*-values. *p*-Value was considered significance when it was less than or equal to 0.05 (^∗^*p* ≤ 0.05, ^∗∗^*p* ≤ 0.01).

The impact of environmental conditions on the microbiota was studied using the enriched conditions as described previously (Wassouf et al., 2018). Using 16S rRNA, we measured first the bacterial compositions at phyla and genera levels in EE conditions, and found that alpha diversity based on Shannon–Weaver index (SWI) was slightly reduced but did not reach to a significant level in both the cecum and colon (Figure 3A). At phylum level, there was no change in the FBR (Supplementary Figures S2B,C, S3B,C). The beta diversity, using the PCoA analysis was recruited and no difference was observed between SNCA-TG and WT in EE conditions, respectively (Supplementary Figures S5A,B). We further performed an explorative analysis based on the 28 most abundant bacterial genera (more than 1% abundance) and representing more than ∼88−92% of the total bacteria sequenced in both the cecum and colon (Figure 3B), similar as in the EE conditions. We identified 5 bacterial genera (*Tangfeifania, Sphingobacterium, Bacteroides, Barnesiella, Prolixibacter*; in decreasing order of *p*-value; student’s *t*-test) in the cecum, and 12 bacterial genera (*Roseburia, Anaerotruncus, Anaerosporobacter, Tannerella, Propionibacterium, Tangfeifania, Lachnoclostridium, Holdemanella, Brevibacterium, Fastidiosipila, Quinella, Succinivibrio*; in decreasing order of *p*-value; student’s *t*-test) in the colon which were different percentage wise in the gut microbiota of SNCA-TG mice in EE conditions (Figures 3B,C). Out of 17 significantly different abundance genera, only *Tangfeifania* genus was commonly present in both the cecum and colon. *Lactobacillus*, the most abundantly occurring genus, was reduced in SNCA-TG mice compared with WT in EE as displayed in the SE conditions, however, it did not reach the significance level (Figure 3B). Moreover, it is worthy to note that a fold-change differences in percentage of *Lactobacillus* increases in SNCA-TG EE compared with SE when data normalized with WT in each environment conditions in both the cecum (0.27 TG_SE/WT_SE to 0.58 TG_EE/WT_EE; fold change = 2.1) and colon (0.52 TG_SE/WT_SE to 0.87 TG_EE/WT_EE; fold change = 1.6) (Figures 2B, 3B).

### EE Condition Affects the Bacterial Abundance

**FIGURE 4:**
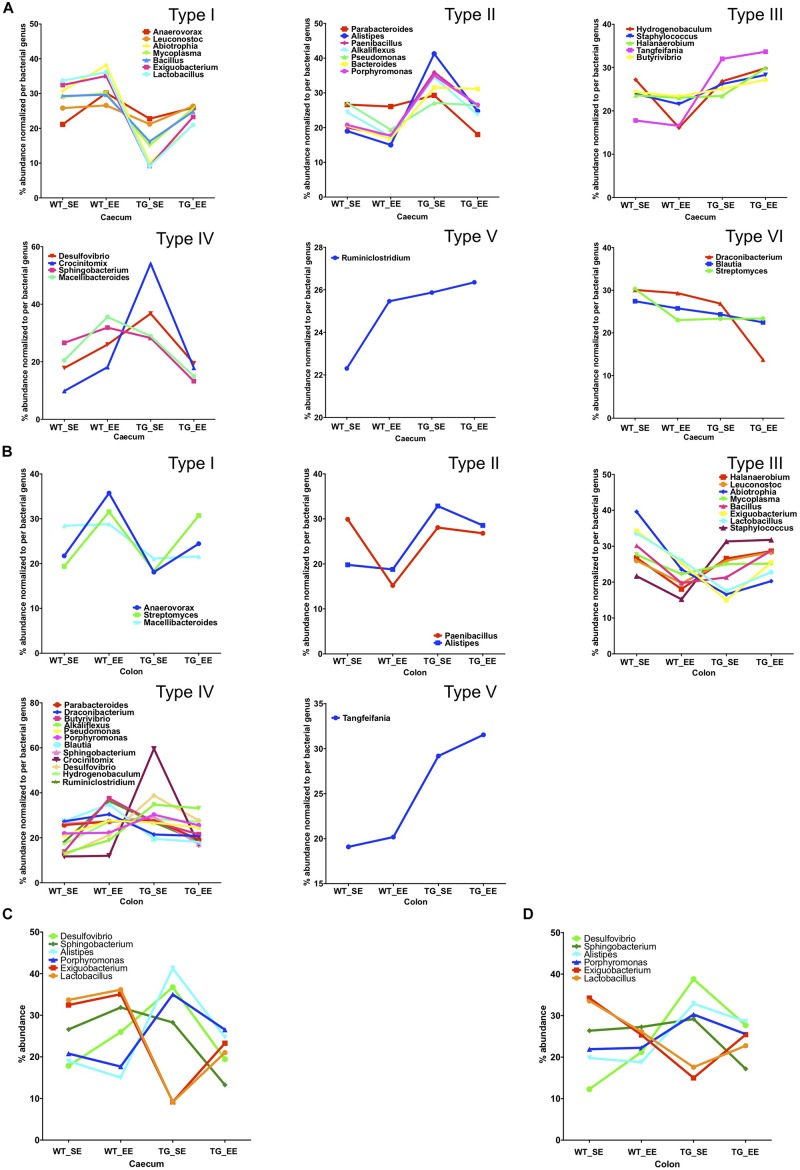
Enriched environment conditions promote the growth of anti-inflammatory bacteria. Comparison of SE and EE conditions using clustering analysis with the MEGEN-CE tool. **(A)** Bacterial phyla were categorized into 6 different types (Type I-VI) for the cecum. **(B)** Bacterial phyla were categorized into 6 different types (Type I-V) for the colon and Type VI was missing in the colon for the 32/540 bacterial genera. **(C)** Bacterial abundance was normalized for each bacterium for SE and EE conditions, 6 bacterial genera were chosen for the cecum which have two different types (Type I and IV) of abundance. No major change in average abundance was between WT SE and EE conditions. **(D)** Whereas, EE conditions tended to normalize the bacterial abundance in SNCA-TG mice. In the colon, for both the WT and SNCA-TG, EE conditions tended to normalize the bacterial abundance.

We calculated the Z-score and clustered bacterial genera for the cecum and colon in the SE and EE conditions at 12 months age for the WT and SNCA-TG groups (Figure 3D and Supplementary Figures S6, S7). We found that the Z-score for the bacterial abundance was higher in the cecum compared with the colon and that SE and EE conditions have the distinct bacterial signature pattern. Further, we measured the alpha diversity using the SWI but found no significant difference between SE and EE conditions (using One-way ANOVA and Tukey *Post hoc* test) in the cecum or colon (Supplementary Figure S8A). We characterized the most abundant bacteria (28 genera) in the cecum and colon of WT and SNCA-TG mice in both SE and EE environments (Supplementary Figures S8A,B). Based on the abundance pattern normalized to the environmental conditions, all bacteria were characterized into 6 different groups for the cecum and 5 different groups for the colon (Figures 4A,B). Furthermore, we standardized percentage bacterial abundance as estimated for SE and EE conditions, and found that EE conditions were able to decrease the percentage gap in the SNCA-TG mice in both the cecum and the colon for the 6 bacterial genera (Figures 4C,D). However, in the WT cecum it was almost similar under SE and EE conditions and it was decreased in the colon in the case of EE compared with SE conditions (Figure 4C).

### EE State Causes Less Inflammatory Reactions in the Feces of SNCA-TG Mice

**FIGURE 5:**
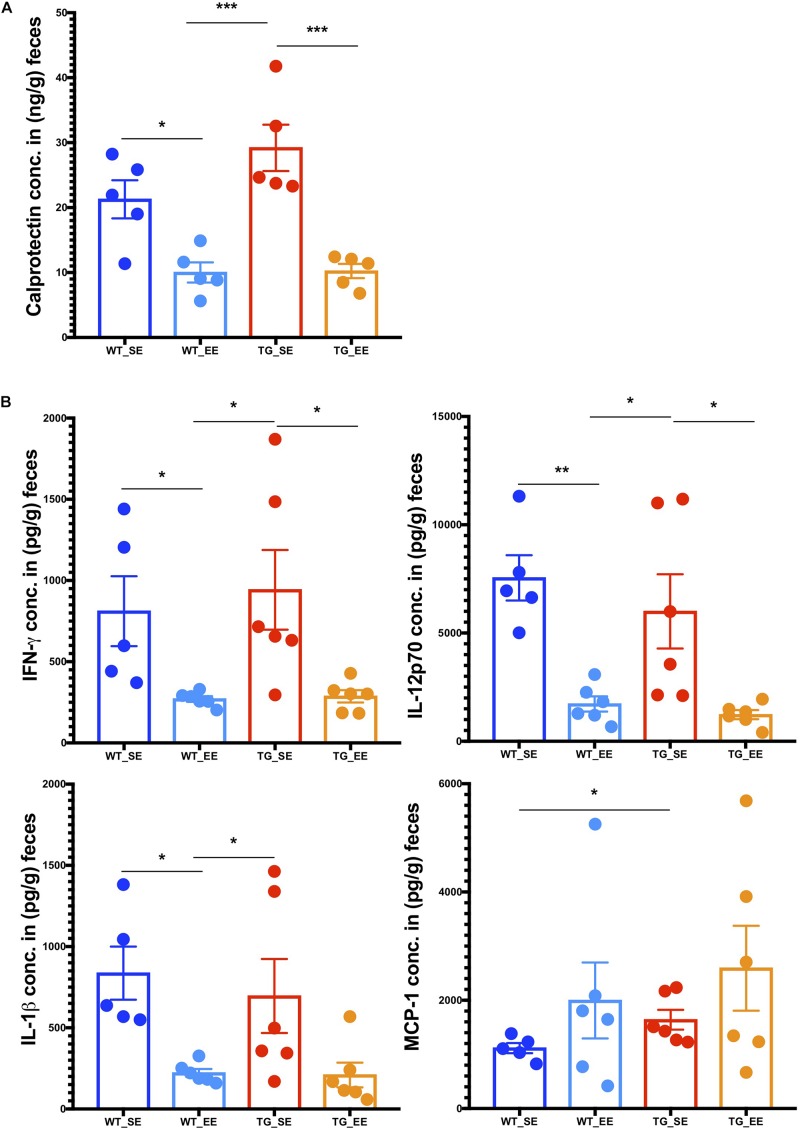
Enriched environment condition dampens the production of inflammatory calprotectin protein and pro-inflammatory cytokines in the feces. **(A)** The colon fecal S100A8/S100A9 (Calprotectin, MRP 8/14) protein was estimated using ELISA. Calprotectin levels were significantly different between SE and EE for the WT and SNCA-TG mice, respectively. One-way ANOVA and a *post hoc* Tukey test was performed to find the significance levels between different groups (SE and EE for WT and SNCA-TG). **(B)** The pro-inflammatory cytokines IFN-γ, IL-12p70, IL-1β, and MCP-1 were estimated using the flow cytometry based-multiplex cytokine assay. IFN-γ and IL-12p70 were significantly different between SE and EE for the WT and SNCA-TG mice, respectively. IL-1β was significantly different between WT SE and EE condition, however, SNCA-TG EE condition tended to have lower IL-1β cytokine, however, no statistical significance was reached. MCP-1 was statistically significant in the SE state between WT and SNCA-TG, however, in EE state it was tended to higher (no statistical difference). All the data are presented in means ± SEM of *n* = 4–5 feces samples per group. *p*-Value was considered significant if it was less than or equal to 0.05 (^∗^*p* ≤ 0.05, ^∗∗^*p* ≤ 0.01, ^∗∗∗^*p* ≤ 0.001) using One-way ANOVA and a *post hoc* Tukey test or/and Student’s *t*-test unpaired *t*-test.

Previous studies in inflammatory bowel disease suggested that the inflammatory calprotectin protein is a virtuous marker of inflammation in the gut (Lehmann et al., 2015; Lee, 2016; Walsham and Sherwood, 2016). Calprotectin is secreted by the neutrophils, macrophages and epithelial cells in the gut lumen as a result of inflammation in the colon (Lehmann et al., 2015). Recent studies in PD patients also suggested that calprotectin levels in the feces were much higher in patients compared with healthy controls (Schwiertz et al., 2018). It appears that calprotectin could be an inflammatory detection marker of PD pathology in the feces of patients (Schwiertz et al., 2018) or possibly even in rodent models as described in this study. Therefore, we measured calprotectin expression from the digesta obtained from the colon from SE and EE conditions in WT and SNCA-TG mice. In SE conditions, SNCA-TG mice had higher levels of calprotectin compared with WT, however, it did not reach the significance level (Figure 5A). On the other hand, in the case of EE conditions (both WT and SNCA-TG mice) calprotectin levels were significantly lower compared to SE conditions. Moreover, SNCA-TG mice have significantly less calprotectin in EE compared with SE conditions (Figure 5A), signifying that EE conditions reduce inflammatory reactions in the gut lumen. Using the LEGENDplex mouse inflammation panel 13-plex, we measured the feces inflammatory cytokines and chemokines and observed that only MCP-1 was significantly upregulated in WT and SNCA-TG group for the SE condition (Figure 5B). EE conditions have no effect on MCP-1, though it tended to be higher in both WT and SNCA-TG EE condition. However, EE was able to significantly reduce inflammatory cytokines such as IFN-γ, IL-12p70 in both the genotypes (WT and SNCA-TG) compared with SE conditions (Figure 5B) as described earlier in the rat (Marashi et al., 2003). While other pro-/anti-inflammatory cytokines (IL-1α/β, TNF-α, IL-23, GM-CSF, IFN-β, IL-17, IL-27, IL-6, and IL-10) tended to be lower in EE compared with SE conditions in SNCA-TG, however, the difference was not at a significant level (Figure 5B and Supplementary Figure S9). Thus, overall the data suggested that EE conditions tended to lower the amount of inflammatory cytokines.

### Enriched Environment Dampens the Inflammatory Genes in the Colon of SNCA-TG Mice

**FIGURE 6:**
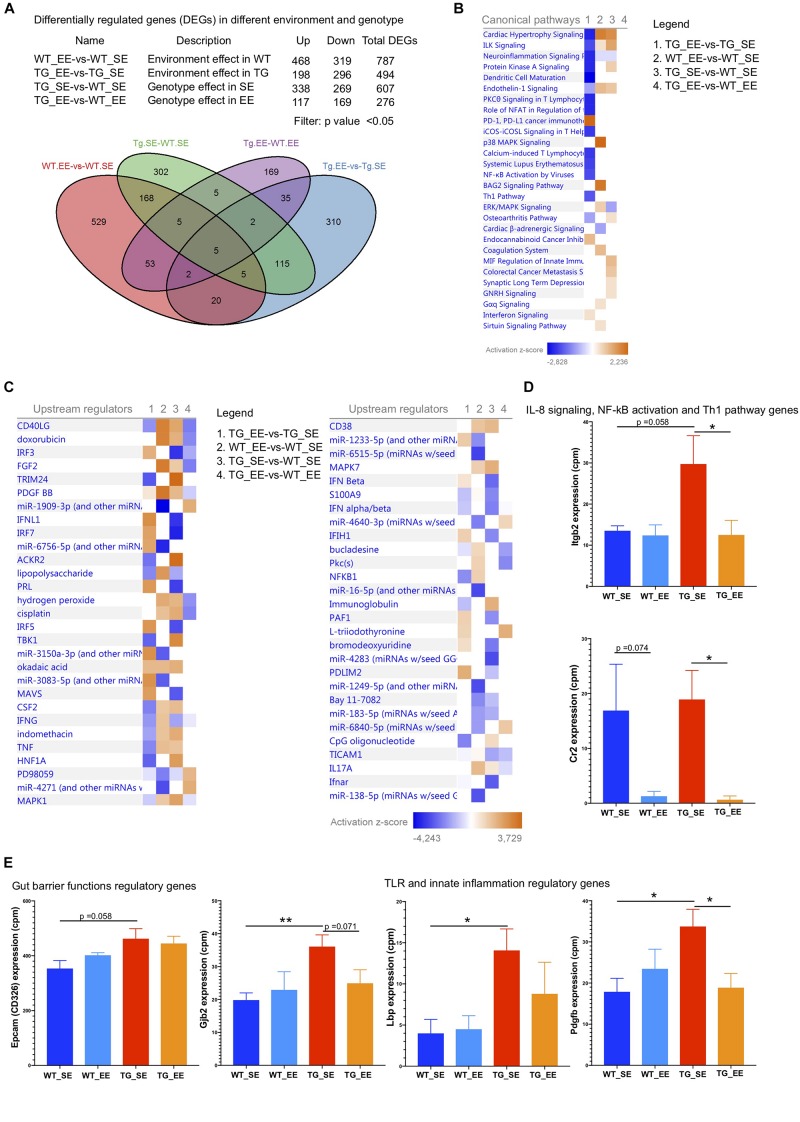
Enriched environment reduces the inflammatory and other signaling pathway genes. **(A)** The differential gene regulations in between the different genotypes and environment. Several genes were upregulated/downregulated based on the four different comparisons including WT_EE-vs-WT_SE, TG_EE-vs-TG_SE, TG_SE-vs-WT_SE and TG_EE-vs-WT_EE. Venn diagram showed the common genes in different environment and genotypes. **(B)** Canonical pathways for the four different comparisons 1. TG_EE-vs-TG_SE, 2. WT_EE-vs-WT_SE, 3. TG_SE-vs-WT_SE and 4. TG_EE-vs-WT_EE based on Ingenuity pathway analysis (IPA). IPA suggested that most of the genes with inflammation pathways including neuroinflammation signaling, protein kinase A signaling, dendritic cells maturation, PKC signaling in T lymphocytes, NFAT in regulation of immune cells, iCOS-iCOSL signaling, NF-kB activation by viruses and Th1 pathways were downregulated in TG (normalized with SE state) compared with WT (normalized with SE state) during enriched conditions. Only PD-1, PD-L1 cancer immunotherapy was upregulated in enriched environment TG animals. **(C)** Most of the inflammatory upstream mediators/regulators were downregulated in TG (EE-vs-SE) compared with WT (EE-vs-SE) state. **(D)** IL-8 signaling, NF-kB activation and Th1 pathways genes (Itgb2 and Cr2) were significantly downregulated in EE conditions in TG group. **(E)** Gut barrier functions regulatory genes (Epcam and Gjb2) were tended to be upregulated in SE conditions in SNCA-TG compared with WT and EE state tended to have reduced expression. Similarly, TLR and innate inflammatory regulatory genes (Lbp and Pdgfb) were significantly upregulated in SNCA-TG compared with WT in SE state and enriched environment downregulated both the genes in SNCA-TG (EE-vs-SE), however, effect was not prominent in WT (SE-vs-EE). All the data are presented in means ± SEM of *n* = 4–5 feces samples per group. *p*-Value was considered significant if it was less than or equal to 0.05 (^∗^*p* ≤ 0.05, ^∗∗^*p* ≤ 0.01) using One-way ANOVA and a *post hoc* Tukey test or/and Student’s *t*-test unpaired *t*-test.

Reduced inflammation in the feces of both WT and SNCA-TG genotypes in EE compared with SE conditions prompted us to investigate whether changes in the microbiome could modulate the signature of the genes in the colon tissues (host-pathogen interactions). Furthermore, previous study also suggested that an EE condition is able to reduce the inflammatory phenotype in the brain of SNCA-TG mice (Wassouf et al., 2018). Thus, we performed the RNA-seq of the colon samples from SE and EE conditions for both the genotypes (WT and SNCA-TG) to discover the effect of environmental-induced changes in the gut microbiome have on the host phenotype and physiology. Our RNA-seq data showed that EE is able to regulate many genes in both the genotypes (WT and SNCA-TG) compared with the SE state (Figure 6A). Many genes were commonly dysregulated in between the environment and genotype (Figure 6A). Using IPA, we tried to identify the genes involved in various canonical pathways for the different genotypes and environment (TG_EE-vs-TG_SE, WT_SE-vs-WT_SE, TG_SE-vs-WT_SE and TG_EE-vs-WT_EE). We found that most of the pathways were down regulated including ILK signaling, neuroinflammation signaling, dendritic cell maturation, PKC signaling in T cells, NFAT regulation, NF-kB activation, Th1 pathway, PD-1, PD-L1 cancer immunotherapy whereas endocannabinoid cancer inhibitory pathway and interferon signaling were upregulated in TG_EE-vs-TG_SE compared with WT_EE-vs-WT_SE or TG_SE-vs-WT_SE conditions (Figure 6B). Many upstream regulators such as TNF, CSF2, IFN-γ, NF-kB1, S100A9, LPS also appeared to have a negative score for the TG_EE-vs-TG_SE compared with WT_SE-vs-WT_SE, TG_SE-vs-WT_SE (Figure 6C). Moreover, individual gene analysis was also performed based on the IPA pathways for the different comparison groups (Supplementary Tables S1–S5). IL-8, NF-kB and Th1 signaling pathway gene such as ‘integrin beta 2’ (Itgb2) was tended to higher in SNCA-TG in SE condition compared with WT and it was nearly significant (*p*-value = 0.058), however, the EE condition was able to significantly reduce the expression of Itgb2 (Figure 6D). Complement receptor 2 (Cr2) gene, which also participates in the IL-8 signaling and NF-kB activation by viruses was significantly downregulated in EE SNCA-TG compared with SE conditions (Figure 6D). The non-motor symptoms such as gut permeability and constipation were frequent in the PD patients (Felice et al., 2016), therefore, we also explored the genes involved in the tight junctions and gut barrier functions and found that the epithelial cell adhesion molecule (Epcam or CD326) was upregulated in SNCA-TG genotype compared with WT in the standard environment. However, EE has no effect on both genotypes including WT and SNCA-TG (Figure 6E). The Gap junction protein, beta 2 (Gjb2) was significantly upregulated in SNCA-TG compared with WT in SE and EE is able to reduce this gene expression nearly at significance level (Figure 6E). Genes involved in TLR (Lps bind protein; Lbp) and innate immunity (platelet derive growth factor, B polypeptide) were upregulated in SNCA-TG compared with WT in SE condition, however, both the genes were downregulated in SNCA-TG in the EE compared to EE condition (Figure 6E). Moreover, genes involved in the inflammation, neuroinflammation, and intracellular signaling cascades were affected mostly in SNCA-TG genotype in EE conditions rather WT (Supplementary Figure S10). Our transcriptomics data revealed that the EE appeared to have a bigger impact on SNCA-TG mice. Overall, RNA-seq transcriptomics data from the colon tissues suggested that the EE condition was able to dampen the inflammatory genes, and this could be the due to change in the microbiome composition.

## Discussion

To study and understand the impact of the gut microbiome in the pathophysiology of PD in patients, complex methodologies using large study groups over a long range of time would be required to eliminate factors such as body mass index, age, exposure to antibiotics, diet or ethnicity. Moreover, it would also be critical to find patients in the preclinical stage not experiencing any non-motor (such as gastrointestinal, sleep disorders) or motor symptoms. In the current state, studying microbiome is based on static studies, meaning that continuous measurement in a controlled environment is extremely challenging to perform in patient cohorts. From this perspective, we are convinced that studying the clear genetic form of Synucleinopathy is providing a more robust datasets which are simpler to interpret due to the limited amount of confounding effects. Moreover, in the context of immune response, inbred animal models kept in a Specified Pathogen Free (SPF) environment present a similar advantage than for the microbiome study. Last but not least, genetic models present a reproducible evolution of the disease allowing pre-symptomatic analysis which cannot be performed on a sporadic disorder like PD. To tackle these problems, rodent models such as mice or rats are an excellent alternative and the way forward to identify and perform functional and mechanistic research on host-microbe interactions as they allow manipulation of the genome, environment, and gut microbiome composition (Nguyen et al., 2015).

In this study, we are reporting that the microbiome is influenced by the expression of human SNCA in our PD mouse model. Importantly, this study also shows that changes in the gut microbiota composition is observed with an increased inflammatory milieu in the gut in SE conditions in 12 months (older) SNCA-TG age group animals, no difference observed in the microbiome diversity and composition in 6 months (young) age group animals (WT and SNCA-TG). Interestingly, a decrease of *Lactobacillus* observed in our study correlates with a recent report in PD patients and appears to be associated with motor impairment (Scheperjans et al., 2015; Unger et al., 2016). We suggest that these changes may be the consequence of the presence or the accumulation of human α-Syn in the GIT of this animal model rather than the cause of the accumulation. In our study, animals from both genotypes were maintained separately in a conventional standard environment and this change in the gut microbiome abundance could be due to ‘after birth’ colonization of pups by microbiota (also termed maternal effects). However, multiple studies have highlighted that the individuality in gut microbiota composition is more affected by environmental effects such as cohort or litter size than by family (Hsiao et al., 2013). To address this question, we kept the animals in the enriched environmental conditions (Wassouf et al., 2018) and found that indeed enriched conditions are able to affect the gut microbiome composition and inflammation in SNCA-TG mice. Therefore, our studies suggest that the environment plays an important role in the modulation of the microbiome composition in the mouse model despite any potential maternal effects.

Furthermore, it is not really surprising that the microbiota α-diversity in our animal SNCA-TG model was not different from WT animals as histological staining did not reveal any known acute inflammatory phenotype. Transcriptomics data from the colon revealed that indeed enriched environment (enriched housing conditions, enhanced sensory stimulation and co-housing, etc.) reduces the inflammatory genes which could be involved in various canonical inflammatory pathways. Our data points out that the “host-microbiome-environment” could play a pivotal role in PD progression as most of the transcriptomics changes related with inflammation occurred in the SNCA-TG mice model in enriched environment. It would have been interesting to test, if metabolism, immunity or hormones would have been modulated in the gut of SNCA-TG mice before or after the shift of microbiota to highlight any potential physiological dysregulation of the gut which could have been induced by immunity or metabolism dysfunction. The exact impact of the microbiome in the development of α-Syn accumulation in the SNCA-TG animals should now be investigated using germ-free conditions or applying broad-spectrum antibiotics. However, uses of antibiotics or germ-free conditions have their own limitation to manipulate the bacterial diversity and maintenance of the host physiology (Langdon et al., 2016). Here, in the current study, we used the enriched environment as a direct intervention to manipulate the bacterial diversity. Earlier studies in the cancer mouse model also suggested that EE environment likely underlie the profound improvement in the survival of colon-tumor bearing mice (Bice et al., 2017; Fuller et al., 2018). Henceforth, enriched environment could be vital to module different diseases including neurodegeneration. Moreover, future studies in PD need to point out whether microbiota can influence the expression levels of α-Syn and if the injection of α-Syn fibrils could lead to changes of gut microbial composition similar to the one observed in our animal model.

In summary, we have shown that mice overexpressing the complete human *SNCA* gene present an expression of transgenic protein in muscular and mucosal layers of the large intestine. Our study also shows that these animals present a shift of bacterial genera similar to that observed in PD patients and not related to potential impairment of locomotor activity. All together, we propose that mice overexpressing the human *SNCA* gene represents a good model to study the impact of the gut microbiome in connection with the expression of α-Syn and low-grade inflammation. Further studies in the animal models are required to challenge or manipulate the gut bacteria, in regard to the low-grade inflammation, either by using individual bacteriophages in combination with neurotoxic compounds (6-hyroxydopamine, paraquat, MPTP, etc.) will help us to unravel the Parkinson’s disease pathology including the non-motor as well as motor phenotypes.

## Data Availability Statement

All datasets generated and analyzed for this study are included in the manuscript/Supplementary Files. RNA-seq data used in this study can also be found on publicly available database on NCBI’s Gene Expression Omnibus under GEO accession: GSE137213. The raw 16S rRNA sequencing data set can also be available to other researchers on a reasonable request.

## Ethics Statement

All animal experimental protocols used in this study is strictly adhered to the international standards for the care and use of laboratory animals and were approved by the local Animal Welfare and Ethics committee of the Country Commission Tübingen, state of Baden-Württemberg, Germany (TVA HG 4/11).

## Author Contributions

YS and NC designed the study, performed the research, analyzed the data, made the figures, and wrote the manuscript. ME-H and JA helped with 16S rRNA data analysis and bioinformatics meta data analysis. UK and LQ-M designed, performed, and analyzed the histology. ZW performed the animal experiments. JS-H helped with animal experiments and study design. DH helped with study design and data analyses. OR designed the study, acquired the funding, and wrote the manuscript. All authors read the manuscript, and approved to be co-authors on the manuscript and have substantial contribution in the manuscript.

## Conflict of Interest

The authors declare that the research was conducted in the absence of any commercial or financial relationships that could be construed as a potential conflict of interest.

## Footnotes

1 https://ab.inf.uni-tuebingen.de/software/malt/welcome/

2 https://www.arb-silva.de/documentation/release-128/

## Supplementary Material

FIGURE S1 Human α-Syn expression in GIT. **(A)** Expression of the transgenic human α-Syn protein was investigated by IHC in different parts of the GIT namely stomach, ileum, jejunum, cecum, colon, rectum to be expressed the α-Syn protein. **(B)** Double immunofluorescence staining from the SNCA-TG colon suggests the presence of total α-Syn (human and mouse α-syn: #610786 BD Biosciences) in enteric neurons labeled with the synaptic markers MAP2, the neuronal markers neurofilament (NeuN), the dopaminergic marker TH, and the inflammatory marker GFAP (in 20×). Red and green color-coded staining shows the respective antibodies staining.

FIGURE S2 The gut dysbiosis at phylum level in the cecum. **(A)** The total number of reads, **(B)** % abundance of bacterial phyla, and **(C)** Firmicutes/Bacteroidetes ratio for the cecum.

FIGURE S3 The gut dysbiosis at phylum level in the colon. **(A)** The total number of reads **(B)** % abundance of bacterial phyla, and **(C)** Firmicutes/Bacteroidetes ratio for the colon.

FIGURE S4 Comparison of microbiome diversity for the 6 months SE condition age group (WT_SE-vs-SNCA-TG_SE). **(A)** Total number of sequences read at phylum level for 6-month age group in WT and SNCA-TG, **(B)** F/B ratio for WT and SNCA-TG. **(C)** Comparison of bacterial composition at phylum level in WT and SNCA-TG in the SE for the colon samples. **(D)** Comparison of the α-diversity for WT and SNCA-TG in the SE for the colon samples in WT and SNCA-TG. **(E)** Shannon–Weaver index (SWI) for WT and SNCA-TG for the 6-month age group. **(F)** The percentage abundance of statistically significantly different (0.01–0.3%; lower abundance) the bacterial genera for WT and SNCA-TG in the SE state for the colon samples. **(G,H)** The bacterial composition for the abundantly present bacteria for the WT and SNCA-TG in the SE state for the colon samples. The most abundant bacteria (greater than 1%) were not significantly different between WT and SNCA-TG in the 6-month age group.

FIGURE S5 Principal component analysis (PCoA) of the bacterial genera in the cecum and colon. To find the different group similarity or dissimilarity as well as SE and EE condition in the cecum and colon, PCoA was performed. **(A)** In the cecum, WT and SNCA-TG SE conditions, bacterial genera were differently clustered, however, in the case of EE condition, WT and SNCA-TG, clustering of the bacterial genera were less prominent. **(B)** In the colon, WT and SNCA-TG SE conditions, bacterial genera were not discretely cluster and a similar cluster was also found for the EE conditions.

FIGURE S6 Clustering of the bacterial genera in the cecum. Comparison of SE and EE conditions using clustering analysis using MEGEN-CE tools in the cecum samples.

FIGURE S7 Clustering of the bacterial genera in the colon. Comparison of SE and EE conditions using clustering analysis using MEGEN-CE tools in the colon samples.

FIGURE S8 Comparison of alpha diversity and abundance of the bacterial phyla in SE and EE conditions. **(A)** Comparison of the α-diversity for the WT and SNCA-TG in the SE and EE conditions for the cecum and colon samples. **(B)** The percentage abundance of the bacterial genera for the WT and SNCA-TG in SE and EE conditions for the cecum and colon samples.

FIGURE S9 Pro- and anti-inflammatory cytokines levels in the feces for the WT and SNCA-TG SE and EE conditions. In the EE state SNCA-TG and WT group of animals tended to have reduced levels of pro-inflammatory cytokinesis (TNF-α, IL-1α, IL-23, GM-CSF, IFN-β, IL-17A, IL-27, and IL-6) in the feces. Anti-inflammatory cytokine, in the EE state of SNCA-TG and WT group of animals tended to also have reduced levels.

FIGURE S10 Genes involved in regulation of the inflammation in SNCA-TG and WT during the SE and EE. **(A)** Neuroinflammatory signaling, Th1 pathway and PD-1, PD-L1 immunotherapy pathway gene (Nos2, Ifnar2, Gata3, and Lck) were changed in SNCA-TG (EE-vs-SE). *p*-Value significance has been described in the Figure. **(B)** Other pro- and anti-inflammatory genes regulation in SE-vs-EE conditions in both the genotypes (WT and SNCA-TG). *p*-Value was considered significant if it was less than or equal to 0.05 (^∗^*p* ≤ 0.05, ^∗∗^*p* ≤ 0.01) using One-way ANOVA and a *post hoc* Tukey test or/and Student’s *t*-test unpaired *t*-test.

TABLE S1 Canonical pathway comparisons.

TABLE S2 Upstream regulators.

TABLE S3 TG_EE-vs.-TG_SE.

TABLE S4 WT_EE-vs.-WT_SE.

TABLE S5 TG_SE-vs.-WT_SE.
