# Supplementary material for: Enriched Environmental Conditions Modify the Gut Microbiome Composition and Fecal Markers of Inflammation in Parkinson’s Disease
Source: Front Neurosci. 2019 Oct 15;13:1032. doi: 10.3389/fnins.2019.01032 (PMC6842954; doi:10.3389/fnins.2019.01032)
Supplement: FIGURE S1 — Human α-Syn expression in GIT. (A) Expression of the transgenic human α-Syn protein was investigated by IHC in different parts of the GIT namely stomach, ileum, jejunum, cecum, colon, rectum to be expressed the α-Syn protein. (B) Double immunofluorescence staining from the SNCA-TG colon suggests the presence of total α-Syn (human and mouse α-syn: #610786 BD Biosciences) in enteric neurons labeled with the synaptic markers MAP2, the neuronal markers neurofilament (NeuN), the dopaminergic marker TH, and the inflammatory marker GFAP (in 20×). Red and green color-coded staining shows the respective antibodies staining. [file Data_Sheet_1.PDF]

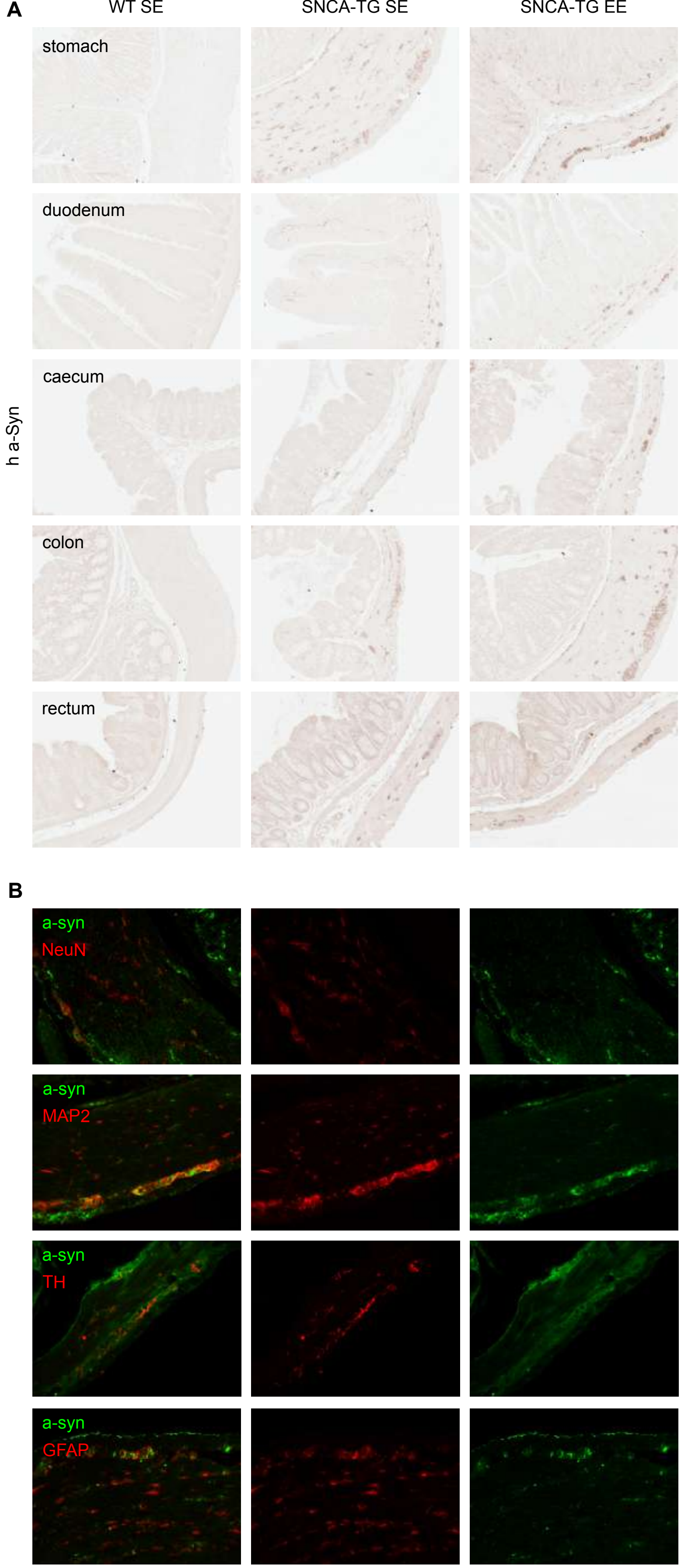
A
WT SE
SNCA-TG SE
SNCA-TG EE
h a-Syn
stomach
duodenum
caecum
colon
rectum
B
a-syn
NeuN
a-syn
MAP2
a-syn
TH
a-syn
GFAP

Suppl. Fig. 1

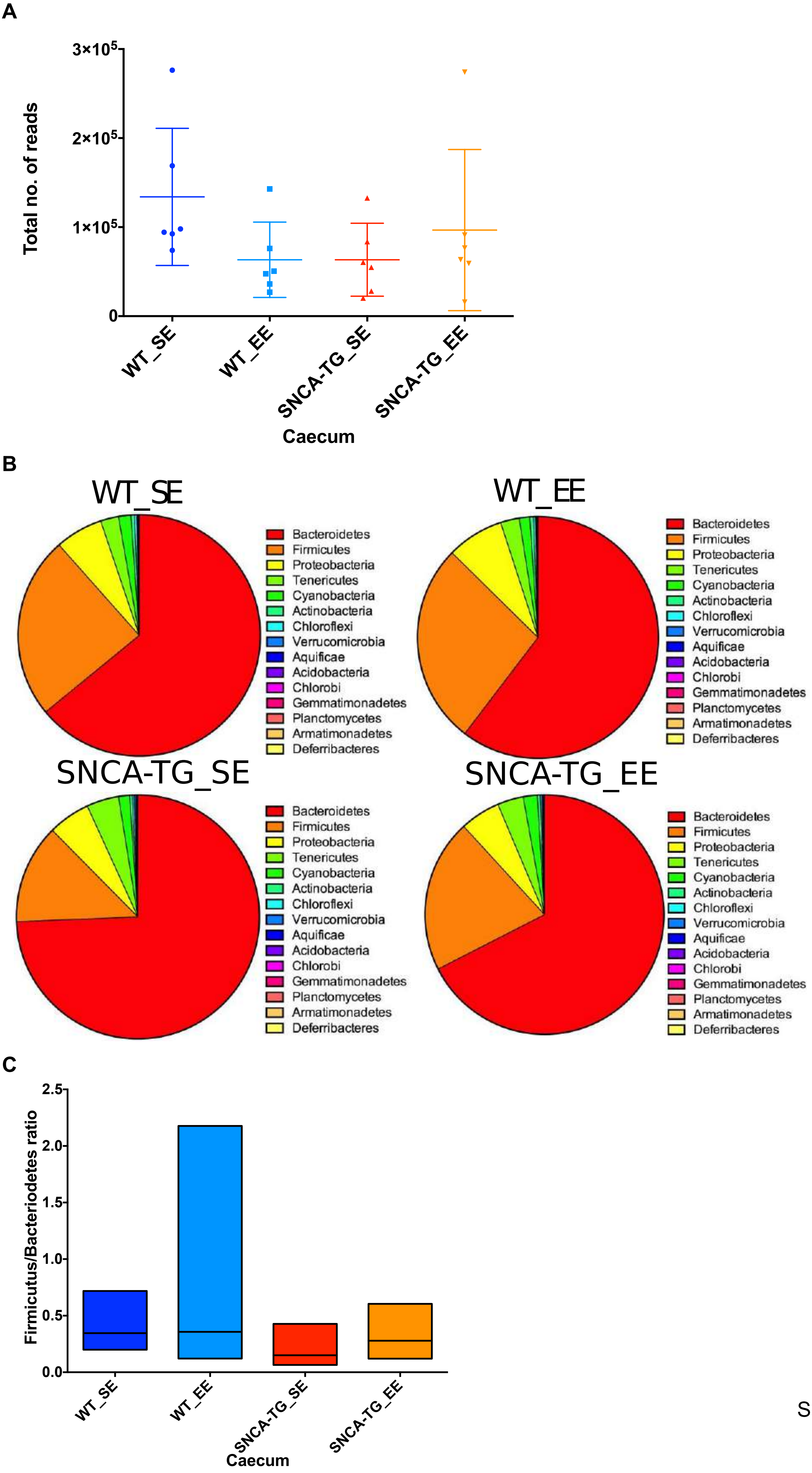
A
Total no. of reads
3×10⁵
2×10⁵
1×10⁵
0
WT_SE
WT_EE
SNCA-TG_SE
SNCA-TG_EE
Caecum
B
WT_SE
WT_EE
SNCA-TG_SE
SNCA-TG_EE
Bacteroidetes
Firmicutes
Proteobacteria
Tenericutes
Cyanobacteria
Actinobacteria
Chloroflexi
Verrucomicrobia
Aquificae
Acidobacteria
Chlorobi
Gemmatimonadetes
Planctomycetes
Armatimonadetes
Deferribacteres
C
Firmicutus/Bacteriodetes ratio
2.5
2.0
1.5
1.0
0.5
0.0
WT_SE
WT_EE
SNCA-TG_SE
SNCA-TG_EE
Caecum

Suppl. Fig. 2

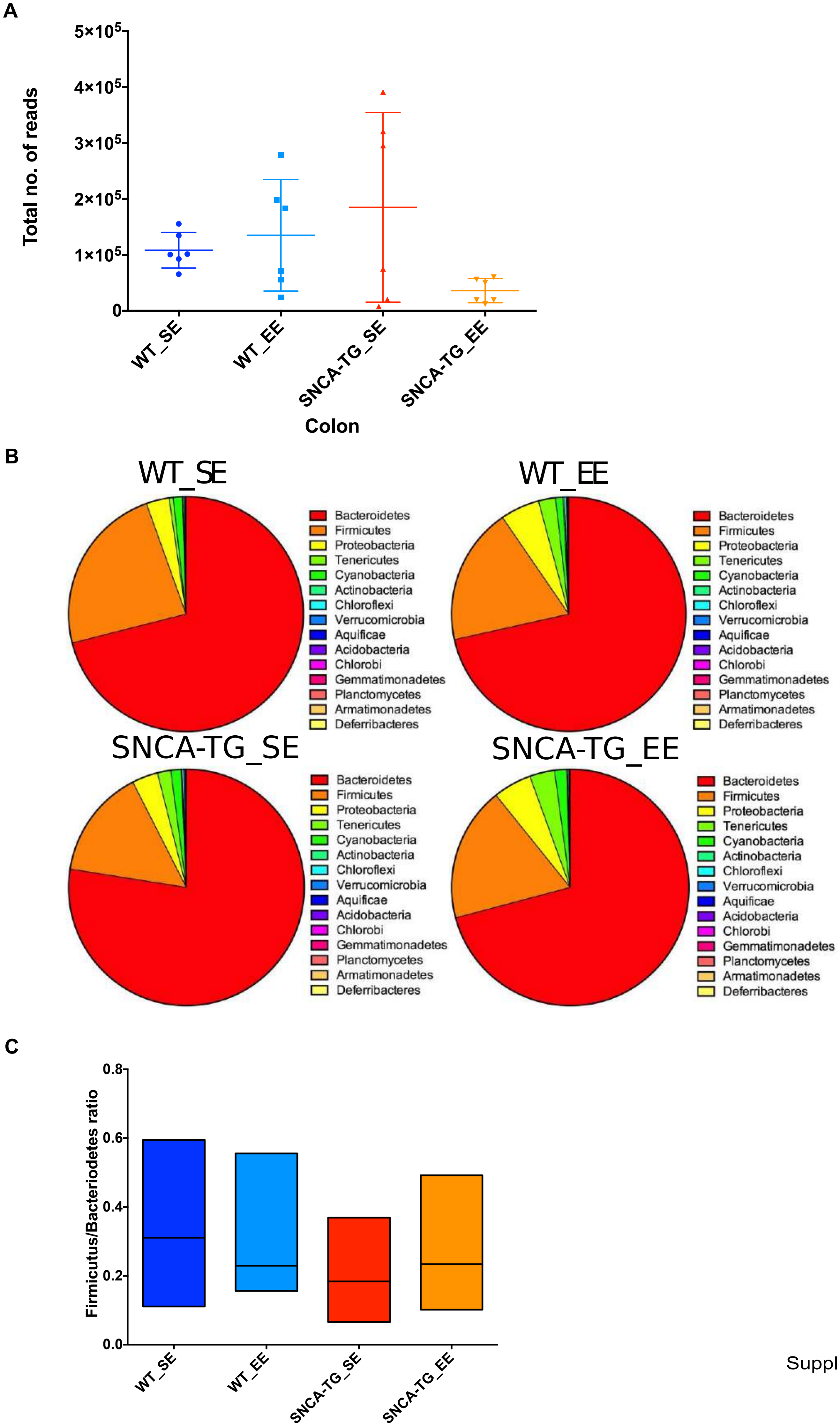
A
Total no. of reads
5×10^5
4×10^5
3×10^5
2×10^5
1×10^5
0
WT_SE
WT_EE
SNCA-TG_SE
SNCA-TG_EE
Colon
B
WT_SE
WT_EE
SNCA-TG_SE
SNCA-TG_EE
Bacteroidetes
Firmicutes
Proteobacteria
Tenericutes
Cyanobacteria
Actinobacteria
Chloroflexi
Verrucomicrobia
Aquificae
Acidobacteria
Chlorobi
Gemmatimonadetes
Planctomycetes
Armatimonadetes
Deferribacteres
C
Firmicutus/Bacteriodetes ratio
0.8
0.6
0.4
0.2
0.0
WT_SE
WT_EE
SNCA-TG_SE
SNCA-TG_EE

Suppl. Fig. 3

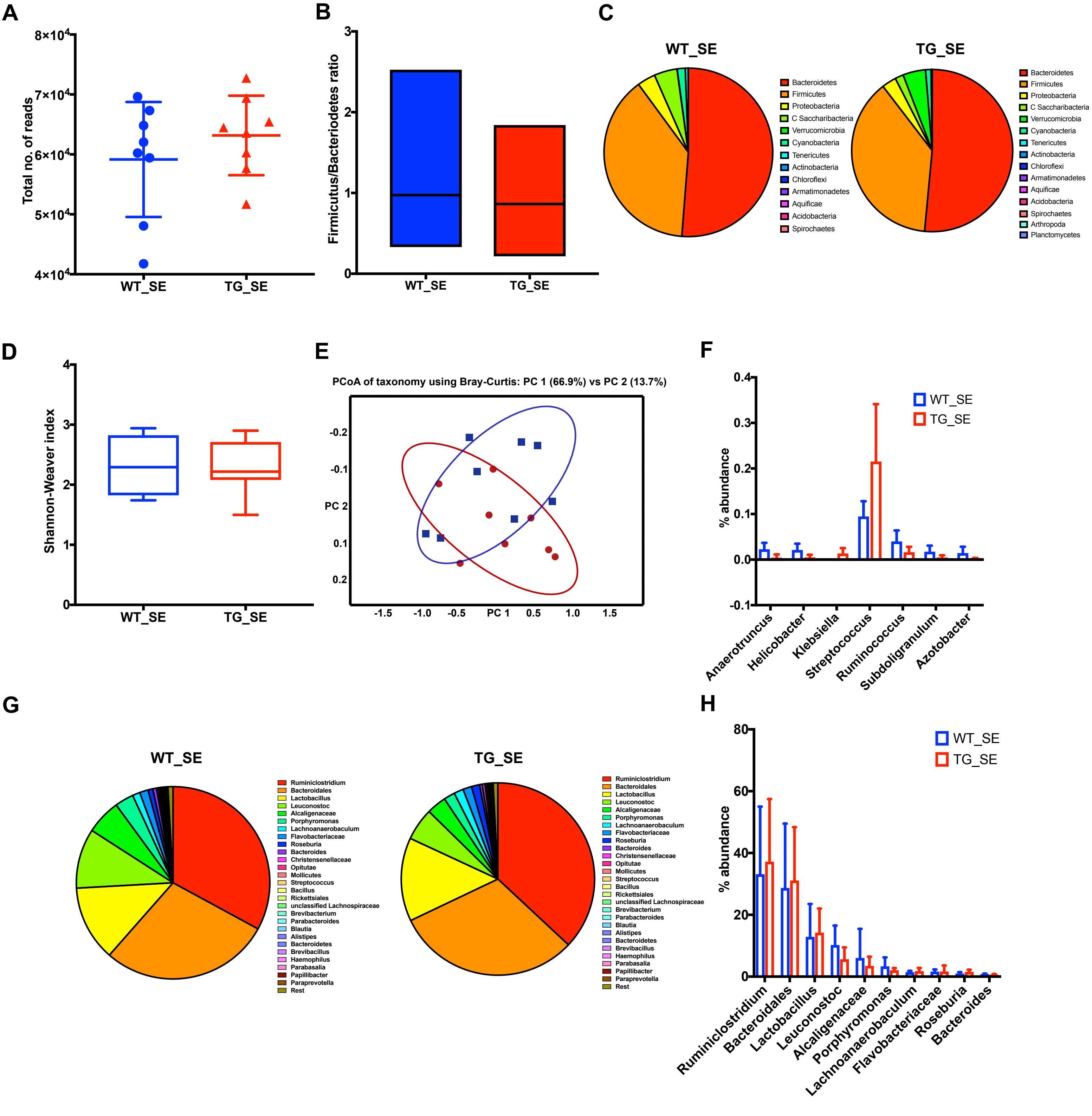
A
8×10^4
7×10^4
6×10^4
5×10^4
4×10^4
Total no. of reads
WT_SE
TG_SE
B
Firmicutus/Bacteriodetes ratio
0
1
2
3
WT_SE
TG_SE
C
WT_SE
TG_SE
Bacteroidetes
Firmicutes
Proteobacteria
C Saccharibacteria
Verrucomicrobia
Cyanobacteria
Tenericutes
Actinobacteria
Chloroflexi
Armatimonadetes
Aquificae
Acidobacteria
Spirochaetes
Arthropoda
Planctomycetes
D
Shannon-Weaver index
0
1
2
3
4
WT_SE
TG_SE
E
PCoA of taxonomy using Bray-Curtis: PC 1 (66.9%) vs PC 2 (13.7%)
-0.2
-0.1
PC 2
0.1
0.2
-1.5
-1.0
-0.5
PC 1
0.5
1.0
1.5
F
% abundance
0.4
0.3
0.2
0.1
0.0
-0.1
WT_SE
TG_SE
Anaerotruncus
Helicobacter
Klebsiella
Streptococcus
Ruminococcus
Subdoligranulum
Azotobacter
G
WT_SE
TG_SE
Ruminiclostridium
Bacteroidales
Lactobacillus
Leuconostoc
Alcaligenaceae
Porphyromonas
Lachnoanaerobaculum
Flavobacteriaceae
Roseburia
Bacteroides
Christensenellaceae
Opitutae
Mollicutes
Streptococcus
Bacillus
Rickettsiales
unclassified Lachnospiraceae
Brevibacterium
Parabacteroides
Blautia
Alistipes
Bacteroidetes
Brevibacillus
Haemophilus
Parabasalia
Papillibacter
Paraprevotella
Rest
H
% abundance
80
60
40
20
0
WT_SE
TG_SE
Ruminiclostridium
Bacteroidales
Lactobacillus
Leuconostoc
Alcaligenaceae
Porphyromonas
Lachnoanaerobaculum
Flavobacteriaceae
Roseburia
Bacteroides

Suppl. Fig. 4

A

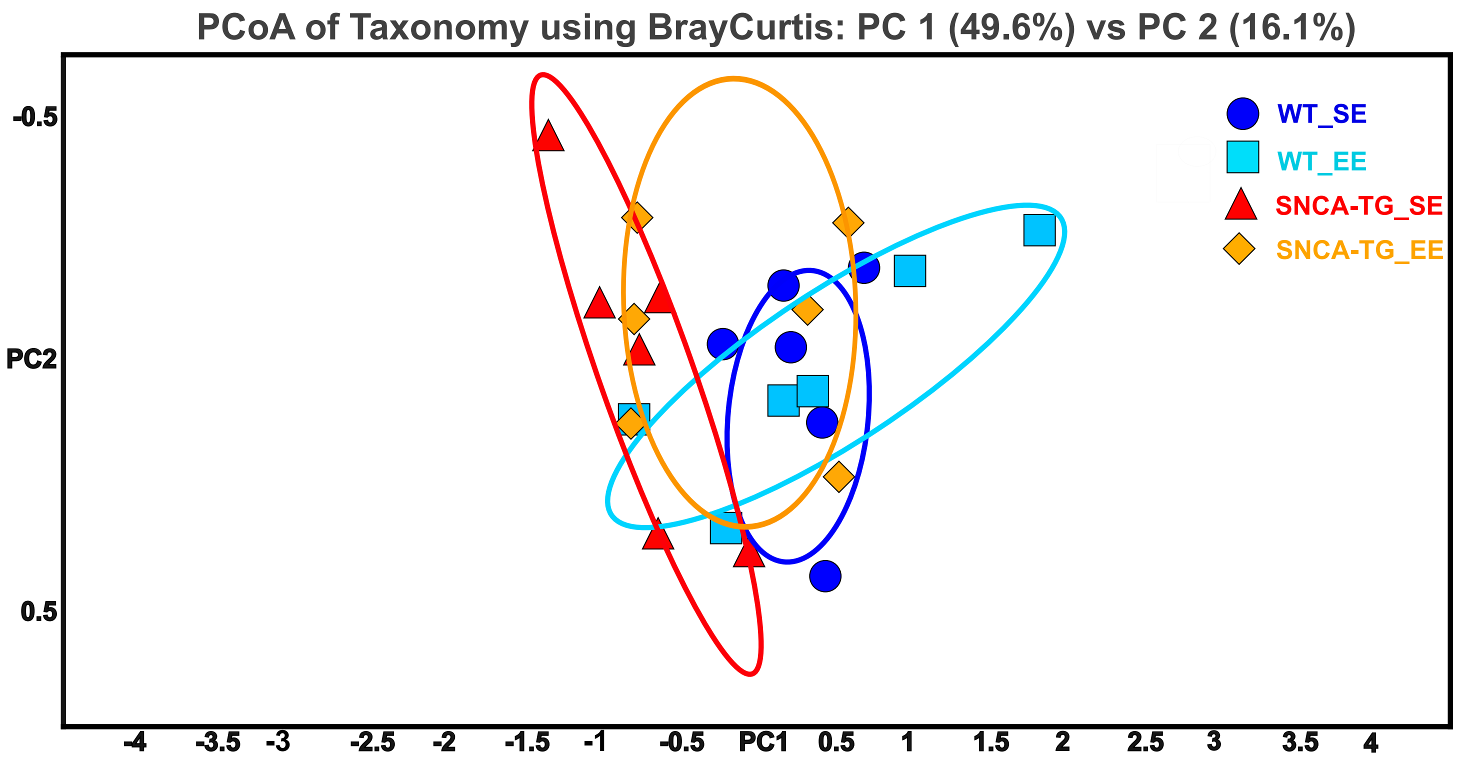

Caecum

B

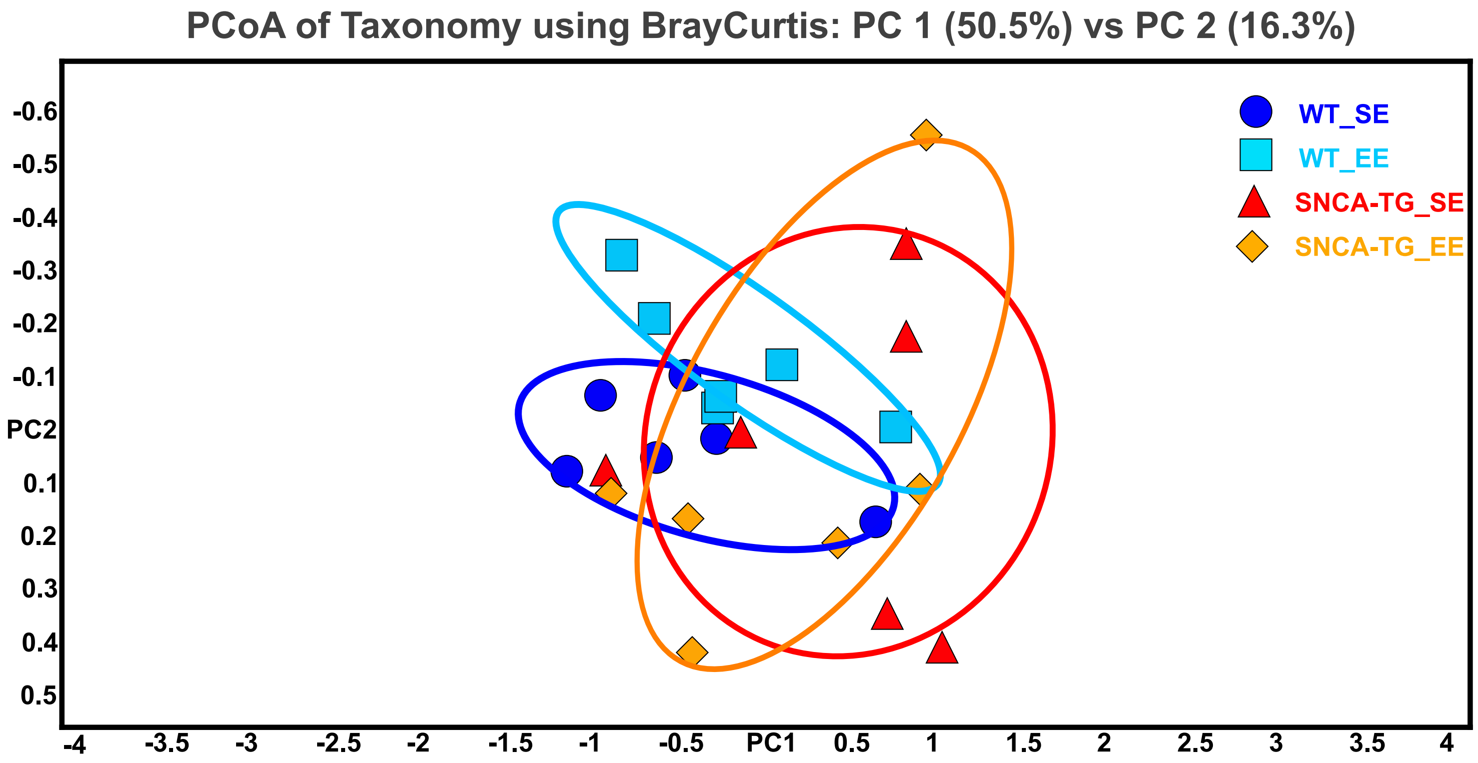

Colon

Suppl. Fig. 5

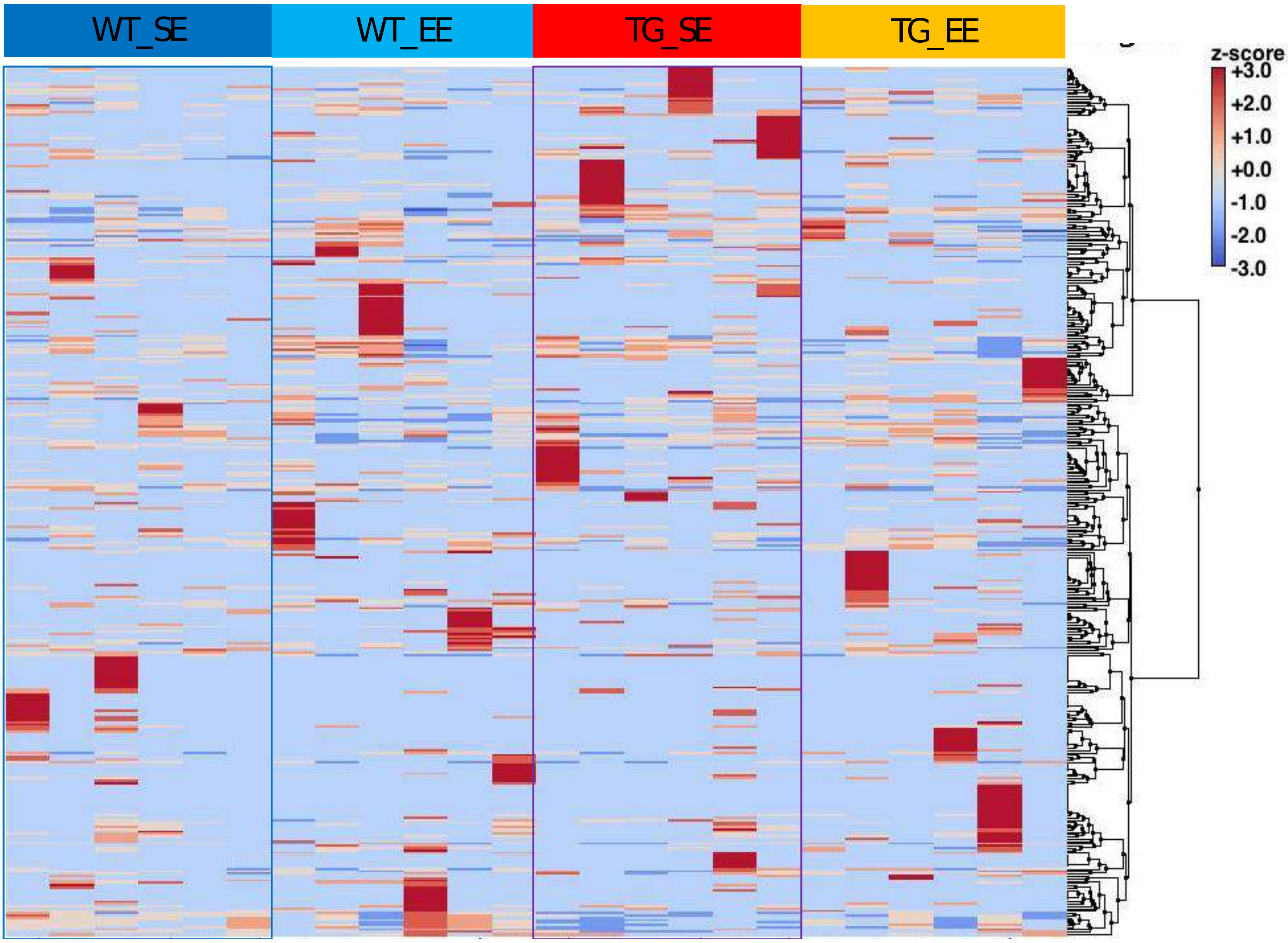

Suppl. Fig. 6

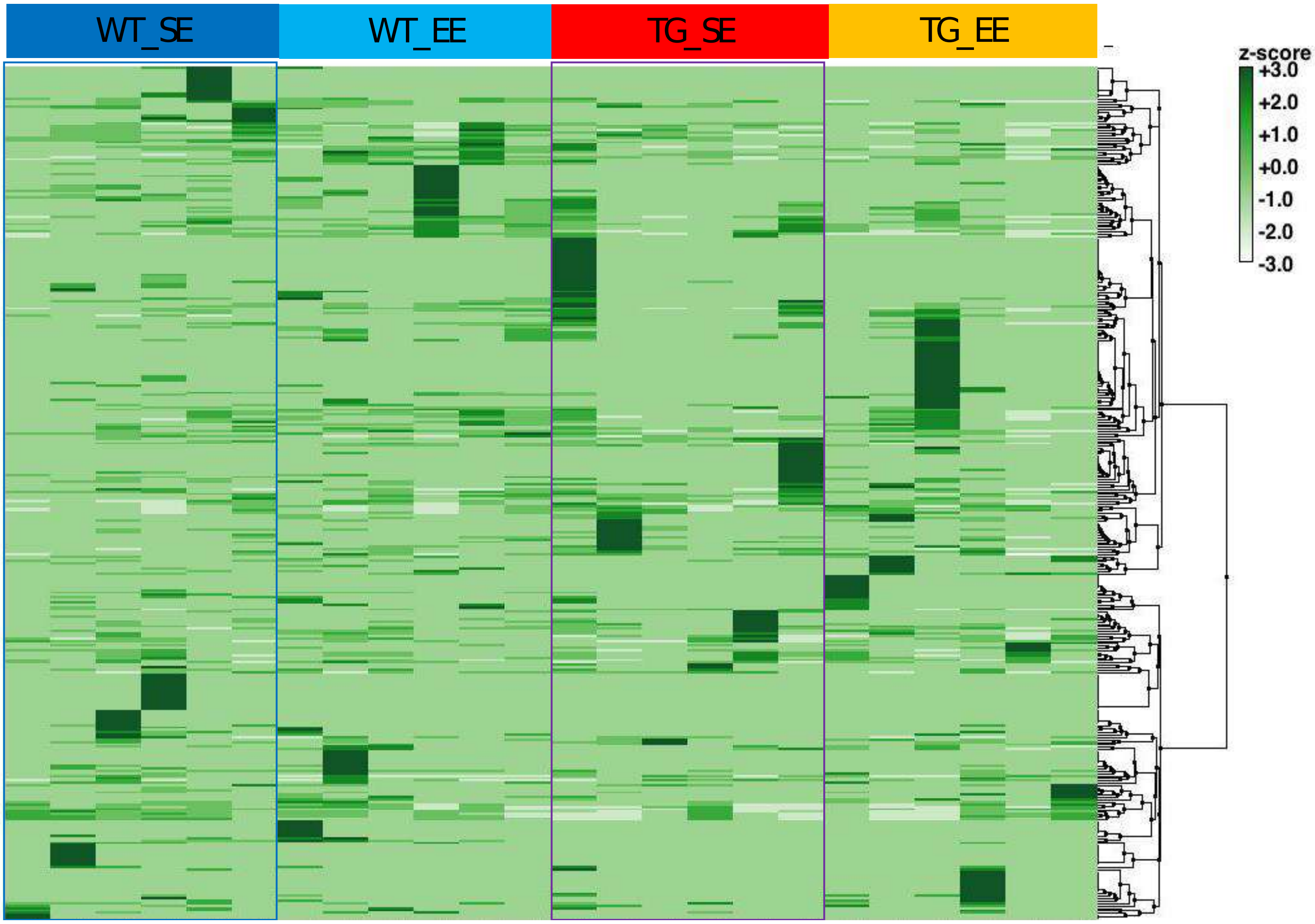

Suppl. Fig. 7

A

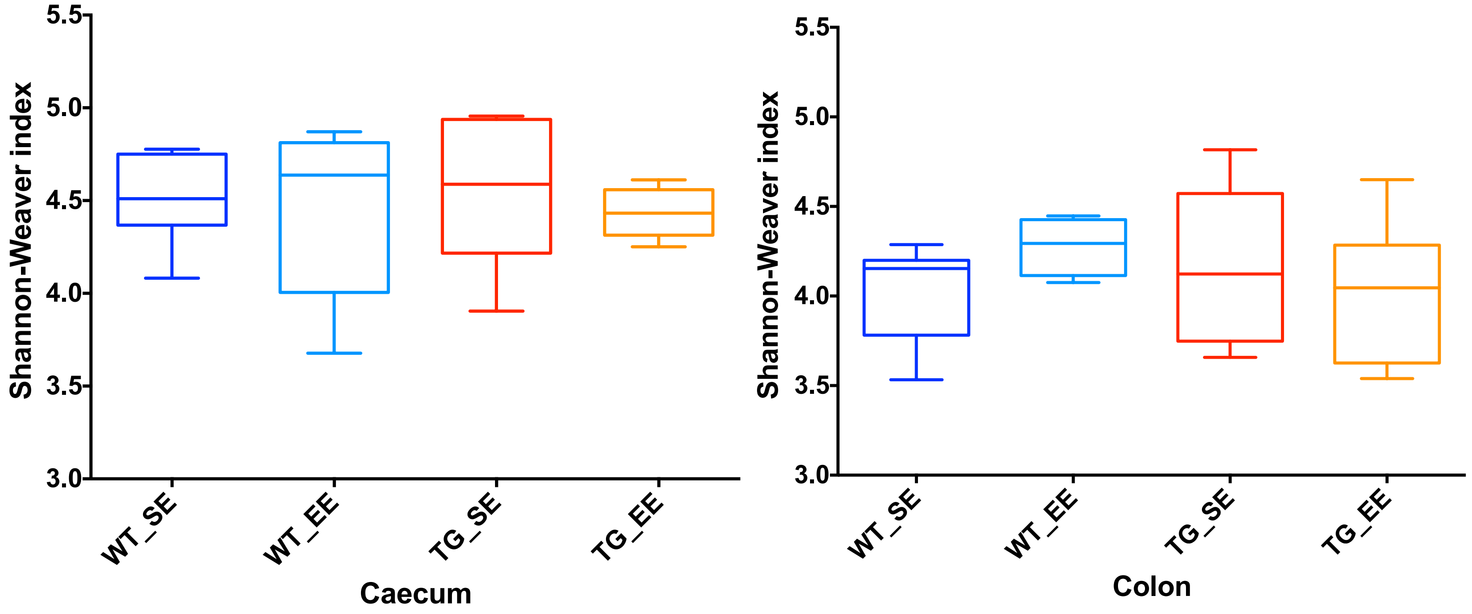

B

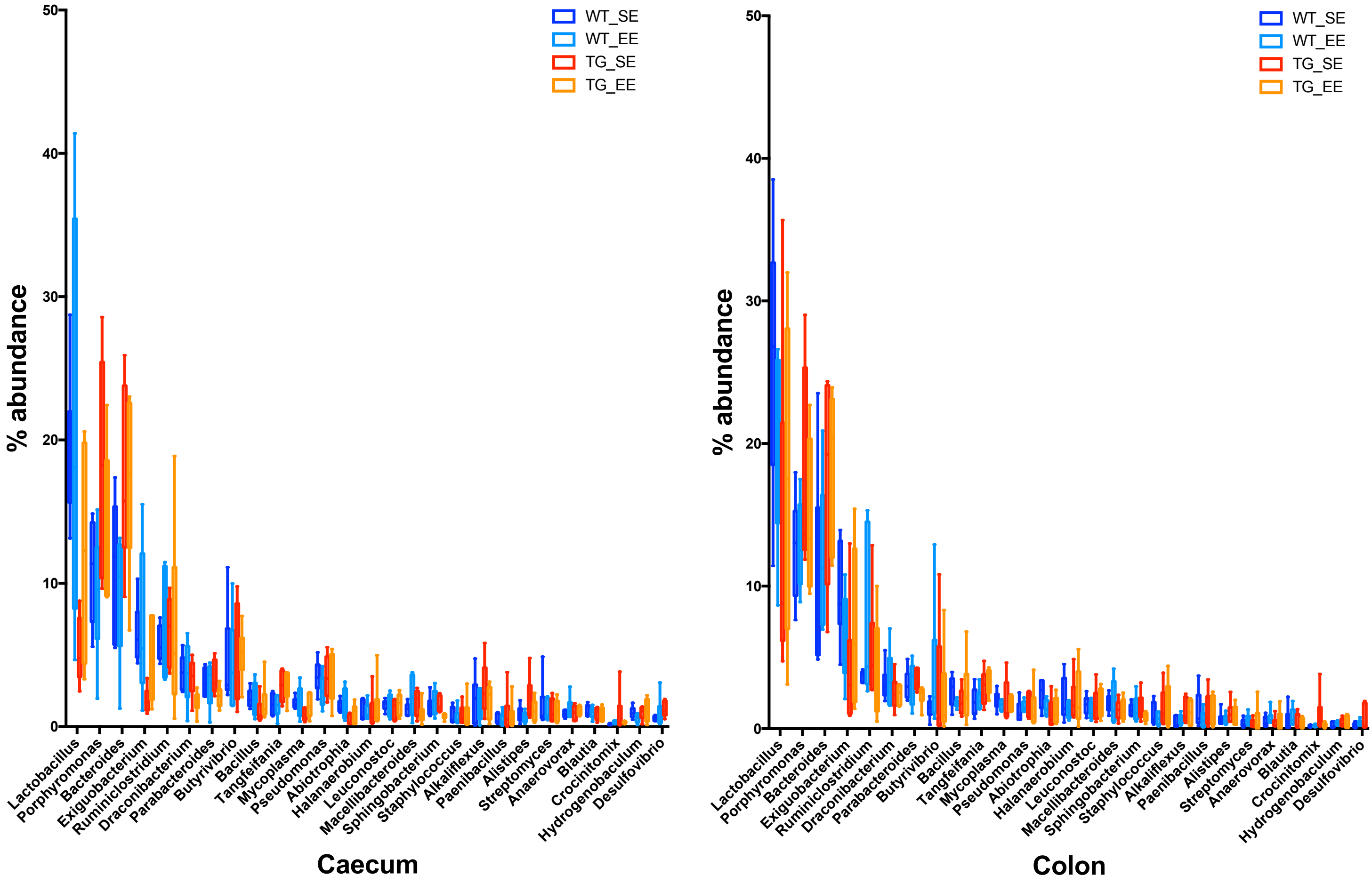

Suppl. Fig. 8

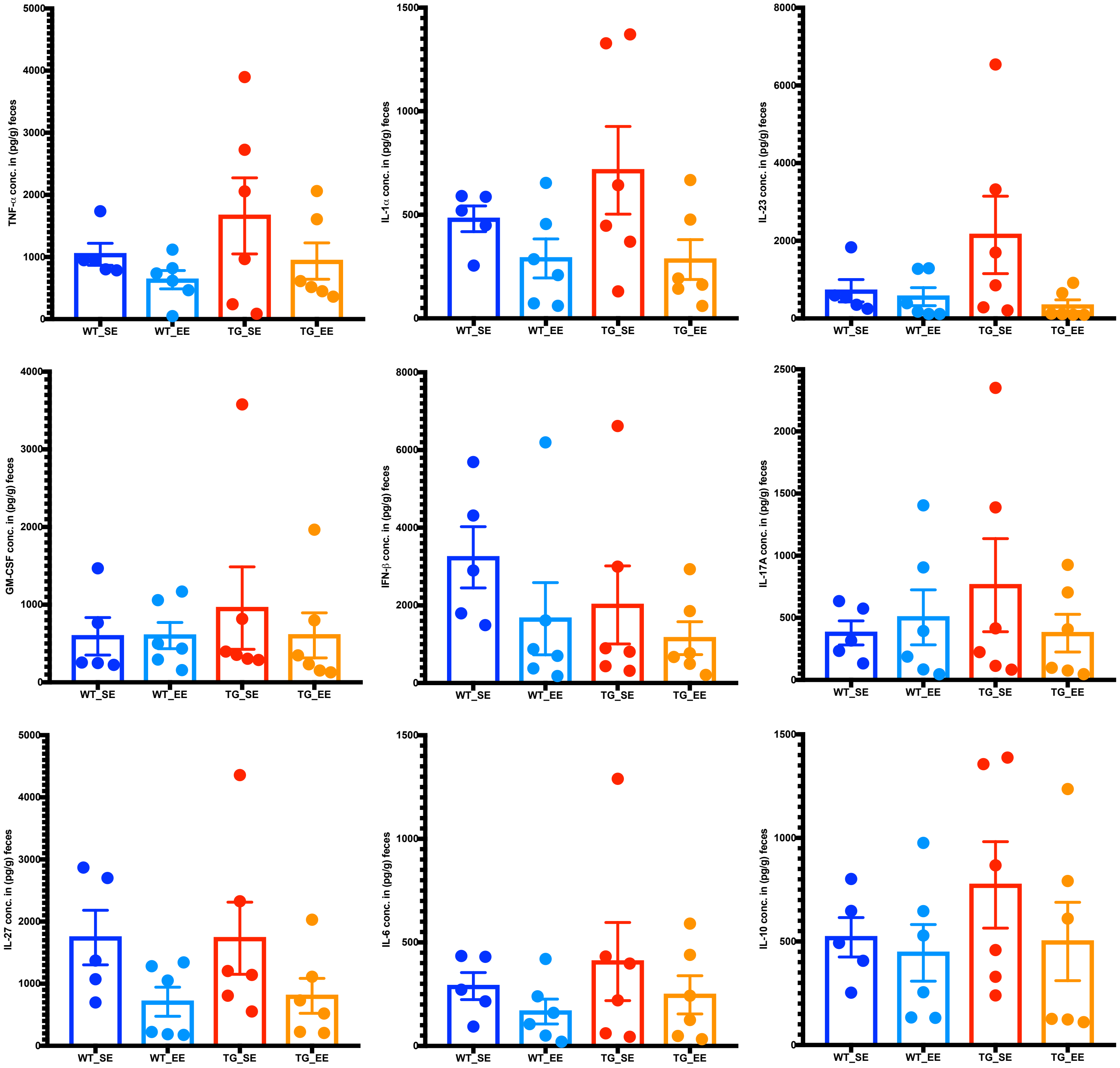
TNF-α conc. in (pg/g) feces
0
1000
2000
3000
4000
5000
IL-1α conc. in (pg/g) feces
0
500
1000
1500
IL-23 conc. in (pg/g) feces
0
2000
4000
6000
8000
GM-CSF conc. in (pg/g) feces
0
1000
2000
3000
4000
IFN-β conc. in (pg/g) feces
0
2000
4000
6000
8000
IL-17A conc. in (pg/g) feces
0
500
1000
1500
2000
2500
IL-27 conc. in (pg/g) feces
0
1000
2000
3000
4000
5000
IL-6 conc. in (pg/g) feces
0
500
1000
1500
IL-10 conc. in (pg/g) feces
0
500
1000
1500
WT_SE
WT_EE
TG_SE
TG_EE

Suppl. Fig. 9

A

Neuroinflammatory signaling, Th1 pathway and PD-1, PD-L1 immunotherapy pathway genes

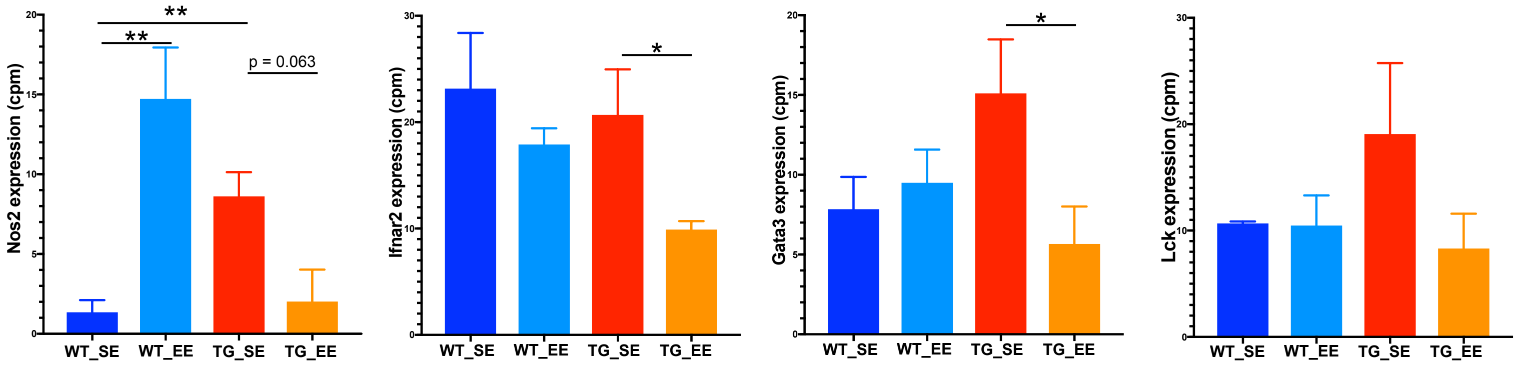

B

Other genes involved in pro/anti-inflammatory signaling pathway

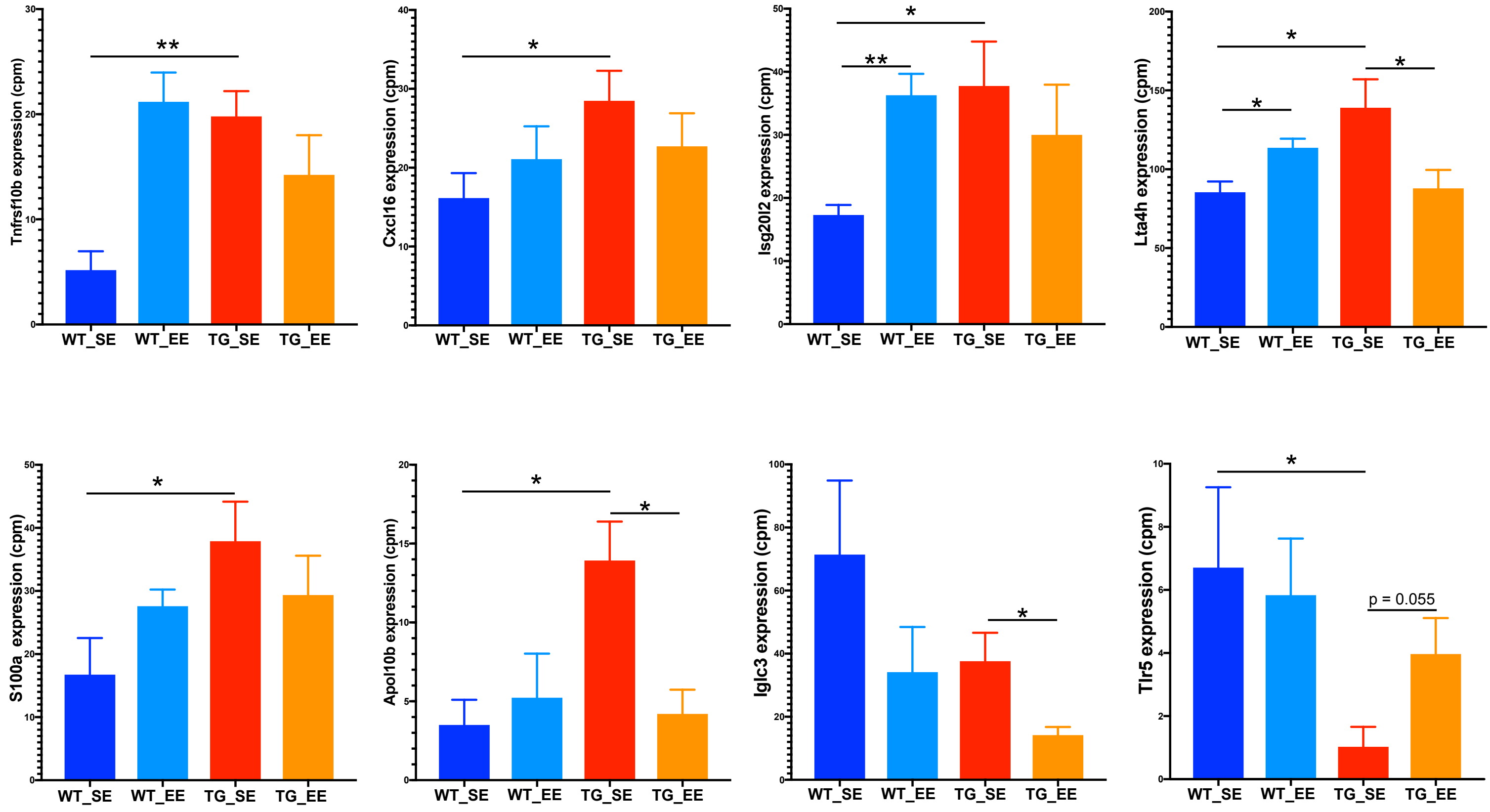

Suppl. Fig. 10

Table 1: Canonical pathways comparisons

**Canonical Pathways**	**TG_EE-TG_SE**	**WT_EE-WT-SE**	**TG_SE-WT_SE**	**TG_EE-WT_EE**
Cardiac Hypertrophy Signaling (Enhanced)	-2.53	1.89	1.667	N/A
ILK Signaling	-2	0.447	1.342	N/A
Neuroinflammation Signaling Pathway	-2.449	-0.378	-0.378	N/A
Protein Kinase A Signaling	-2.449	0	0.707	N/A
Dendritic Cell Maturation	-2.828	N/A	N/A	N/A
Endothelin-1 Signaling	-1	1	0.816	N/A
PKCθ Signaling in T Lymphocytes	-2.449	N/A	N/A	N/A
Role of NFAT in Regulation of the Immune Response	-2.449	N/A	N/A	N/A
PD-1, PD-L1 cancer immunotherapy pathway	2.236	N/A	N/A	N/A
iCOS-iCOSL Signaling in T Helper Cells	-2.236	N/A	N/A	N/A
p38 MAPK Signaling	N/A	2.236	N/A	N/A
Calcium-induced T Lymphocyte Apoptosis	-2.236	N/A	N/A	N/A
Systemic Lupus Erythematosus In T Cell Signaling Pathway	-2.121	0	N/A	N/A
NF-κB Activation by Viruses	-2	N/A	N/A	N/A
BAG2 Signaling Pathway	N/A	2	N/A	N/A
Th1 Pathway	-1.89	N/A	N/A	N/A
ERK/MAPK Signaling	N/A	0.707	-1	N/A
Osteoarthritis Pathway	-1	0	0.447	N/A
Cardiac β-adrenergic Signaling	N/A	-1	0	N/A
Endocannabinoid Cancer Inhibition Pathway	1	N/A	N/A	N/A
Coagulation System	N/A	1	N/A	N/A

Table 2: Upstream regulators

Upstream Regulators	TG_EE-TG_SE	WT_EE-WT-SE	TG_SE-WT_SE	TG_EE-WT_EE
CD40LG	-1.807	3.09	2.198	-2.172
IRF3	1.931	N/A	-3.456	-1.387
TRIM24	-2.813	N/A	3.729	N/A
ACKR2	-2.333	N/A	3.606	N/A
lipopolysaccharide	-1.996	2.519	-1.42	N/A
PRL	2.547	N/A	-3.212	N/A
IRF5	2.588	N/A	-2.961	N/A
TBK1	-2.621	N/A	2.907	N/A
CSF2	-2.096	1.362	1.632	N/A
IFNG	-1.735	1.48	-1.503	-0.363
indomethacin	-1.537	1.5	2.038	N/A
TNF	-1.989	1.607	1.471	N/A
HNF1A	-2.611	N/A	2.449	N/A
PD98059	-0.476	-2.124	-0.61	1.788
MAPK1	-0.837	1.326	2.311	-0.497
E. coli B5 lipopolysaccharide	-2.545	N/A	N/A	-2.341
ITGB1	-1.246	0.968	1.575	-1.091
POR	2.63	N/A	-2.219	N/A
NFkB (complex)	-2.389	2.445	N/A	N/A
tetradecanoylphorbol acetate	-0.736	2.885	N/A	-1.091
TGFB1	0.938	2.563	1.132	N/A
IRF8	-2.193	N/A	2.409	N/A
beta-estradiol	-0.656	1.412	1.783	0.7
CD40	-0.808	1.417	2.323	N/A
IFNA2	0.854	-0.685	-2.957	N/A
PARP9	2	N/A	-2.433	N/A
MYD88	-1.548	0.589	-0.14	-2.152
IFNA1/IFNA13	1.989	N/A	-2.38	N/A
SIRT1	-1.387	N/A	2.887	N/A
VCAN	1.915	N/A	-2.359	N/A
IL13	-1.211	1.803	0.456	-0.595
Interferon alpha	1.289	0.186	-2.526	N/A
poly rI:rC-RNA	0.595	1.198	-2.191	N/A
IL4	-1.803	0.619	1.07	-0.215
AHR	-0.949	N/A	0.669	-1.991
IL6	-0.09	1.462	0.795	-1.208
STAT3	-0.458	1.454	1.57	N/A
DNASE2	-1.974	N/A	1.498	N/A

cyclosporin A	-1.486	N/A	1.958	N/A
cyclic AMP	-1.428	N/A	N/A	-1.961
Tlr	N/A	2.213	1.131	N/A
CpG oligonucleotide	-2.064	N/A	0.878	N/A
TICAM1	-0.192	0.2	-1.47	-1.069
IL17A	N/A	1.706	0.601	-0.586
TLR4	-1.49	1.079	0.287	N/A
IRF1	1.283	N/A	-1.477	N/A
IRF4	-0.478	N/A	2.236	N/A
cytokine	-0.368	0.882	1.376	N/A
IL1B	-0.965	0.612	0.424	-0.472
GATA4	-1.724	0.594	0.063	N/A
IL12 (complex)	-1.247	N/A	1.05	N/A
IL1RN	-0.853	N/A	1.086	0.277
TLR9	0.288	N/A	-0.998	-0.692

Table 3: TG_EE-vs-TG_SE

**Ingenuity Canonical Pathways**	**-log(p-value)**	**Ratio**	**z-score**	**Molecules**
Interferon Signaling	4.68E+00	1.39E-01	0.45	IFIT3,**IFNAR1**,IFNAR2,ISG15,OAS1
Th1 Pathway	4.67E+00	6.50E-02	-1.89	DLL1,**GATA3**,HLA-A,HLA-DMA,HLA-DMB,HLA-DRB5,IFNAR1,ITGB2
Calcium-induced T Lymphocyte Apoptosis	4.44E+00	9.09E-02	-2.24	FCER1G,HLA-A,HLA-DMA,HLA-DMB,HLA-DRB5,**LCK**
Dendritic Cell Maturation	4.20E+00	4.89E-02	-2.83	CREB3L3,FCER1G,HLA-A,HLA-DMA,HLA-DMB,HLA-DRB5,**IFNAR1**,Ighg2b,PLCH2
iCOS-iCOSL Signaling in T Helper Cells	3.18E+00	5.36E-02	-2.24	FCER1G,HLA-A,HLA-DMA,HLA-DMB,**HLA-DRB5**,LCK
PD-1, PD-L1 cancer immunotherapy pathway	2.47E+00	4.67E-02	2.24	**HLA-A**,HLA-DMA,HLA-DMB,HLA-DRB5,LCK
PKCθ Signaling in T Lymphocytes	2.44E+00	3.85E-02	-2.45	**FCER1G**,HLA-A,HLA-DMA,HLA-DMB,HLA-DRB5,LCK
Cardiac Hypertrophy Signaling (Enhanced)	2.19E+00	2.26E-02	-2.53	ADCY7,CSF2RB,Eda,FGF7,IFNAR1,IL21R,IL7R,PDE10A,PLCH2,RPS6KA5,SMPDL3B
Role of NFAT in Regulation of the Immune Response	2.12E+00	3.30E-02	-2.45	FCER1G,HLA-A,HLA-DMA,HLA-DMB,HLA-DRB5,LCK
NF-κB Activation by Viruses	2.11E+00	4.82E-02	-2.00	**CR2**,ITGAL,ITGB2,LCK
Systemic Lupus Erythematosus In T Cell Signaling Pathway	1.85E+00	2.39E-02	-2.12	CREB3L3,FCER1G,HLA-A,HLA-DMA,HLA-DMB,HLA-DRB5,ITGAL,**NOS2**
ILK Signaling	1.45E+00	2.60E-02	-2.00	ACTA1,CREB3L3,**ITGB2,NOS2**,RPS6KA5
Endocannabinoid Cancer Inhibition Pathway	1.33E+00	2.78E-02	1.00	ADCY7,CCNE1,CREB3L3,NOS2
Neuroinflammation Signaling Pathway	1.19E+00	1.99E-02	-2.45	**CREB3L3**,HLA-A,HLA-DMA,HLA-DMB,HLA-DRB5,**NOS2**
Opioid Signaling Pathway	1.06E+00	2.00E-02	-2.00	ADCY7,CREB3L3,FOSB,LCK,RPS6KA5
Endothelin-1 Signaling	1.00E+00	2.14E-02	-1.00	ABHD3,ADCY7,NOS2,PLCH2
IL-8 Signaling	9.21E-01	2.00E-02	-1.00	**CR2**,ITGAX,ITGB2,TEK
Osteoarthritis Pathway	8.51E-01	1.88E-02	-1.00	ALPP,CREB3L3,**ITLN1,NOS2**

Integrin Signaling	8.42E-01	1.86E-02	-2.00	ACTA1,ITGAL,ITGAX,ITGB2
cAMP-mediated signaling	7.80E-01	1.76E-02	-2.00	ADCY7,CREB3L3,PDE10A,SMPDL3B
Protein Kinase A Signaling	7.59E-01	1.50E-02	-2.45	ADCY7,CREB3L3,DUSP10,PDE10A,PLCH2,SMPDL3B
Synaptogenesis Signaling Pathway	4.70E-01	1.28E-02	-1.00	ADCY7,CREB3L3,EPHA5,LCK

Table 4: WT_EE-vs-WT_SE

**Ingenuity Canonical Pathways**	**-log(p-value)**	**Ratio**	**z-score**	**Molecules**
Coagulation System	3.22E+00	1.14E-01	1.00	F13A1,F2R,PLAT,PLAU
BAG2 Signaling Pathway	2.88E+00	9.30E-02	2.00	BAG2,HSPA1A/HSPA1B,Hspa1b,HSPA2
ERK/MAPK Signaling	2.81E+00	4.12E-02	0.71	CREB5,HSPB1,HSPB7,PLA2G4C,PPP1R14B,PPP1R3C,PPP2CB,SRF
p38 MAPK Signaling	1.98E+00	4.24E-02	2.24	CREB5,HSPB1,HSPB7,PLA2G4C,SRF
Sirtuin Signaling Pathway	1.77E+00	2.74E-02	0.45	ARNTL,CRTC2,GADD45G,MT-ATP6,NDUFAF1,NOS2,SDHD,Tomm5
Cardiac β-adrenergic Signaling	1.69E+00	3.57E-02	-1.00	ENPP6,GNG5,PPP1R14B,PPP1R3C,PPP2CB
ILK Signaling	1.68E+00	3.12E-02	0.45	CREB5,FLNC,NOS2,PPP1R14B,PPP2CB,PTGS2
Neuroinflammation Signaling Pathway	1.28E+00	2.33E-02	-0.38	CREB5,HLA-DMB,HMOX1,NOS2,NOX1,PLA2G4C,PTGS2
Cardiac Hypertrophy Signaling (Enhanced)	1.02E+00	1.85E-02	1.89	AGTR1,ENPP6,FGF7,GNG5,HSPB1,HSPB7,PTGS2,SRF,TNFSF12
eNOS Signaling	9.83E-01	2.50E-02	-1.00	CHRM3,HSPA1A/HSPA1B,Hspa1b,HSPA2
Opioid Signaling Pathway	8.30E-01	2.00E-02	1.00	CREB5,FOSB,GNG5,RGS1,SRF
Endothelin-1 Signaling	8.04E-01	2.14E-02	1.00	HMOX1,NOS2,PLA2G4C,PTGS2
Production of Nitric Oxide and Reactive Oxygen Species in Macrophages	7.93E-01	2.12E-02	-1.00	NOS2,PPP1R14B,PPP1R3C,PPP2CB
cAMP-mediated signaling	6.06E-01	1.76E-02	1.00	AGTR1,CHRM3,CREB5,ENPP6
Synaptogenesis Signaling Pathway	5.67E-01	1.60E-02	1.34	CREB5,EPHA2,RASGRP2,STXBP1,SYT5

Table 5: TG_SE-vs-WT_SE

Ingenuity Canonical Pathways	-log(p-value)	Ratio	z-score	Molecules
MIF Regulation of Innate Immunity	3.03E+00	9.52E-02	1.00	NOS2,PLA2G4C,PLA2G6,PTGS2
Endothelin-1 Signaling	1.87E+00	3.21E-02	0.82	ABHD3,ADCY7,NOS2,PLA2G4C,PLA2G6,PTGS2
ILK Signaling	1.82E+00	3.12E-02	1.34	ACTA1,ITGB7,NOS2,PPP1R14B,PPP2CB,PTGS2
GNRH Signaling	1.46E+00	2.89E-02	0.45	ADCY7,CAMK2B,GNG5,MAP3K6,MMP2
Neuroinflammation Signaling Pathway	1.42E+00	2.33E-02	-0.38	ACVR1,IRF7,NOS2,PLA2G4C,PLA2G6,PTGS2,TLR5
Synaptic Long Term Depression	1.34E+00	2.67E-02	0.45	ABHD3,NOS2,PLA2G4C,PLA2G6,PPP2CB
Colorectal Cancer Metastasis Signaling	1.30E+00	2.36E-02	0.82	ADCY7,GNG5,MMP2,NOS2,PTGS2,TLR5
Phospholipase C Signaling	1.28E+00	2.33E-02	1.34	ADCY7,GNG5,IGHG1,Ighg2b,PLA2G4C,PLA2G6
Protein Kinase A Signaling	1.24E+00	2.01E-02	0.71	ADCY7,CAMK2B,CDC26,DUSP10,GNG5,PPP1R14B,PTGS2,SMPDL3B
Cardiac Hypertrophy Signaling (Enhanced)	1.16E+00	1.85E-02	1.67	ACE,ACVR1,ADCY7,AGTR1,CAMK2B,GNG5,MAP3K6,PTGS2,SMPDL3B
Osteoarthritis Pathway	1.14E+00	2.35E-02	0.45	BGLAP,GREM1,ITLN1,NOS2,PTGS2
Synaptogenesis Signaling Pathway	9.67E-01	1.92E-02	0.82	ADCY7,CAMK2B,EPHA2,NRXN2,RASGRP2,THBS2
ERK/MAPK Signaling	8.42E-01	2.06E-02	-1.00	PLA2G4C,PLA2G6,PPP1R14B,PPP2CB
